# Characterization, Biocompatibility and Antioxidant Activity of Hydrogels Containing Propolis Extract as an Alternative Treatment in Wound Healing

**DOI:** 10.3390/ph17050575

**Published:** 2024-04-30

**Authors:** Lindalva Maria de Meneses Costa Ferreira, Yuri Yoshioka Modesto, Poliana Dimsan Queiroz de Souza, Fabiana Cristina de Araújo Nascimento, Rayanne Rocha Pereira, Attilio Converti, Desireé Gyles Lynch, Davi do Socorro Barros Brasil, Edilene Oliveira da Silva, José Otávio Carréra Silva-Júnior, Roseane Maria Ribeiro-Costa

**Affiliations:** 1 Institute of Health Sciences, Federal University of Pará, Belém 66075-110, Brazil; lindalva.costa.ferreira@ics.ufpa.br (L.M.d.M.C.F.); yuriyoshioka742@hotmail.com (Y.Y.M.); carrera@ufpa.br (J.O.C.S.-J.); 2 Institute of Biological Sciences, Federal University of Pará, Belém 66075-110, Brazil; polianadimsan@gmail.com (P.D.Q.d.S.); edilene@ufpa.br (E.O.d.S.); 3 Institute of Technology, Federal University of Pará, Belém 66075-110, Brazil; fabiana.nascimento@ics.ufpa.br (F.C.d.A.N.); davibb@ufpa.br (D.d.S.B.B.); 4 Institute of Collective Health, Federal University of Western Pará, Santarém 68035-110, Brazil; rayanne.pereira@ufopa.edu.br; 5 Department of Civil, Chemical and Environmental Engineering, University of Genoa, Pole of Chemical Engineering, via Opera Pia 15, 16145 Genoa, Italy; converti@unige.it; 6 School of Pharmacy, College of Health Sciences, University of Technology, Jamaica, 237 Old Hope Road, Kinston 6, Jamaica; desiree.gyles@utech.edu.jm

**Keywords:** *Apis mellifera*, natural product, propolis, hydrogel, dressing, cytotoxicity

## Abstract

Hydrogels consist of a network of highly porous polymeric chains with the potential for use as a wound dressing. Propolis is a natural product with several biological properties including anti-inflammatory, antibacterial and antioxidant activities. This study was aimed at synthesizing and characterizing a polyacrylamide/methylcellulose hydrogel containing propolis as an active ingredient, to serve as a wound dressing alternative, for the treatment of skin lesions. The hydrogels were prepared using free radical polymerization, and were characterized using scanning electron microscopy, infrared spectroscopy, thermogravimetry, differential scanning calorimetry, swelling capacity, mechanical and rheological properties, UV-Vis spectroscopy, antioxidant activity by the DPPH, ABTS and FRAP assays and biocompatibility determined in Vero cells and J774 macrophages by the MTT assay. Hydrogels showed a porous and foliaceous structure with a well-defined network, a good ability to absorb water and aqueous solutions simulating body fluids as well as desirable mechanical properties and pseudoplastic behavior. In hydrogels containing 1.0 and 2.5% propolis, the contents of total polyphenols were 24.74 ± 1.71 mg GAE/g and 32.10 ± 1.01 mg GAE/g and those of total flavonoids 8.01 ± 0.99 mg QE/g and 13.81 ± 0.71 mg QE/g, respectively, in addition to good antioxidant activity determined with all three methods used. Therefore, hydrogels containing propolis extract, may serve as a promising alternative wound dressing for the treatment of skin lesions, due to their anti-oxidant properties, low cost and availability.

## 1. Introduction

Hydrogels have a hydrophilic three-dimensional network capable of absorbing large amounts of water [1,2,3]. They can be classified as chemical hydrogels when their structure is characterized by the presence of covalent bonds that are formed by chemical reticulation, by the addition of reticulation agents. They can also be classified as physical hydrogels, when polymeric chains are physically reticulated through non-covalent interactions [4,5]. Hydrogels can be synthesized from natural or synthetic polymers or even by the association of both [6,7]. Although hydrogels prepared from synthetic polymers do not have bioactive properties, which limits their application in the medical area, they have excellent mechanical and hydrophilic properties [6]. Conversely, hydrogels prepared from natural polymers stand out for their biocompatibility, biodegradability and presence of biological molecules [8,9], but may have unsatisfactory mechanical properties.

Among the polymers used to synthesize hydrogels are polyacrylamide, a synthetic polymer, and methylcellulose, a natural polysaccharide derived from cellulose [4,6,10].

Methylcellulose is a hydrophilic poly-hydroxylated polymer, which is soluble in aqueous media and practically insoluble in anhydrous ethanol, ether and acetone [11]. Thanks to its unique gelling properties, it is deemed a promising sustainable material for the preparation of three-dimensional network structures, such as hydrogels [12]. Most methylcellulose gels are applicable to biological systems, when used between ambient and body temperatures [13]. Chemical reticulation produces robust hydrogels with properties that can be controlled at low temperature [11]. Methylcellulose gels can be easily prepared, are safe, non-toxic and have good film-forming properties [12]. The compatibility of these hydrogels with water allows their use in a wide variety of applications, including the production of pharmaceutical, cosmetic and personal hygiene products, food, ceramics and construction materials [13]. However, in addition to having low mechanical resistance, aqueous methylcellulose solutions are thermoreversible, i.e., they revert to liquid solutions after cooling, which limits their technological applications [4,11,12]. This limitation can be minimized by incorporating them into polymeric networks consisting of synthetic polymers such as polyacrylamide [14,15].

Polyacrylamide, a synthetic copolymer widely used in hydrogel synthesis, is stable, non-toxic and non-immunogenic [16]. It is the preferred matrix for the preparation of more complex copolymer hydrogels because of its highly elastic nature that can be easily chemically modified, due to the abundance of functional groups [17]. In addition, it is biocompatible and biodegradable and is used in various hygiene products, wound treatment dressings and the production of contact lenses [17]. Polyacrylamide hydrogels are economical, moldable and can combine with biological materials [16]. Due to the ease of being chemically infused with other elements or compounds, polyacrylamide is widely applied to create innovative polymer systems with unique structure and features for use in wound dressings, biosensors, drug administration, tissue regeneration and cartilage repair [6].

Due to its special properties, such as high swelling capacity, porosity, soft structure, flexibility, chemical durability and biocompatibility, hydrogels have been used in various applications in the food industry, water purification and agriculture industries, as absorbents, in the reconstruction of soft tissues and tissue engineering scaffolding [5,10,18], drug administration, pharmaceutical and personal hygiene products, medical applications such as ligament and tendon prostheses or robotic actuators [7], and obtaining new wound dressings [19]. Wound dressings should absorb excess exudate, provide a moist environment at the wound site and have adequate permeability to oxygen. Since infections slow wound healing, application of natural products with antibacterial, antifungal, antioxidant and anti-inflammatory properties in dressings can be a good alternative to accelerate it [20].

In this context, natural products are considered the main sources of bioactive compounds capable of combating the most diverse pathological conditions [21]. Propolis is a natural product, consisting of a complex mixture of resinous substances, gums and balsamics collected by honey bees in sprouts, flowers and plant exudates, to which the bees add salivary secretions, wax and pollen [22]. Propolis from different regions and locations have such a different chemical composition that more than 300 substances have been identified so far, including phenolic acids, flavonoids, esters, diterpenes, sesquiterpenes, lignans, aromatic aldehydes, aromatic alcohols, fatty acids, vitamins and minerals [23]. These compounds are considered to be responsible for the various pharmacological activities of propolis, such as antibacterial, antioxidant, anti-influenza, antifungal, antileishmanial, antitumoral, analgesic, anti-inflammatory [23] and antipromastigote [24] healing activities. All these properties make this product a viable natural additive for incorporation into biomaterials for application in the most diverse areas [25]. 

The antimicrobial chemical constituents of propolis play a fundamental role in the mechanism of accelerating healing. Propolis has a unique effect on trauma healing, as it acts to relieve pain, acts as an antibacterial, promotes the regeneration of wound tissue, promotes granulation growth and limits the formation of scars [26]. Armed [27] showed in their study evidence of the antibacterial action of propolis in the treatment of diabetic wounds and burns. M. Afonso et al. [28] evaluated the in vitro healing activity of honey samples and propolis extracts on normal human dermal fibroblasts (NHDFs) using the scratch assay and showed that honey and propolis were able to promote cell migration suggesting its healing potential.

Kapare et al. [29] developed a hydrogel formulation with PVA-based Indian propolis with potential for wound healing, which showed promising results for future studies regarding the evaluation of the therapeutic effect in clinical trials. Voss et al. [30] formulated cellulose-based films loaded with propolis and propolis combined with vitamin C, and showed that these were a promising wound healing treatment, by significantly reducing (by more than 50%) bacterial counts, improving wound closure and histological determinants of healing, when tested on diabetic mice. Pillai et al. [31] evaluated the healing potential of Indian propolis extract and ointment on excisional wounds induced in Wistar rat models, and showed promising results by accelerating the healing process in various phases of tissue repair.

Hydrogels resulting from the association of synthetic (polyacrylamide) and natural (methylcellulose) polymers have been developed as a strategy to optimize mechanical and biological processes and expand their application in medical and technological areas [14,15]. Moreover, the incorporation of extracts from natural products into the matrix of hygrogels increases their application potential as active dressings [26]. Several studies have been developed to evaluate the therapeutic potential of polyacrylamide/methylcellulose hydrogels containing different extracts. For instance, Gyles et al. [6] prepared a hydrogel containing *Aloe barbadensis* for application as a bandage in the treatment of chronic skin lesions, while Ferreira et al. [10] demonstrated that a hydrogel containing calendula extract promoted tissue regeneration and accelerated the healing process with reduced production of proinflammatory factors in Wistar rats. Based on this background, we prepared in this study propolis-loaded polyacrylamide and methylcellulose hydrogels to be used as an alternative dressing to treat topical lesions.

## 2. Results

### 2.1. Gas Chromatography-Mass Spectrometry Assay (GC-MS) of Propolis Extract

In the Gas Chromatography analysis, five terpenes were detected, in addition to 14 other compounds. The classes of substances found were ketones, hydrocarbons, alcohols and esters. Table 1 presents the percentage by area of the substances identified in the propolis extract. In this study, triterpenes were identified, corroborating the findings in the literature and the results of NMR assay.

### 2.2. Nuclear Magnetic Resonance Spectrometry (NMR) of Propolis Extract

The propolis extract was analyzed by hydrogen and carbon NMR. In the ^1^H NMR spectrum (Figure S1, Supplementary Materials), the absence of some signals corresponding to important functional groups were observed, such as: carboxylic acid, in which the carbonyl hydrogen (H-COH) is absorbed in a region between 13.2 and 10 ppm, aldehydes that absorb at frequencies greater than 10 ppm, enols (β-diketones) that absorb between 16 and 14 ppm, enols (β-ketoesters) that absorb at 10.5 and 9.5 ppm and sulfonic acid that absorbs between 12 and 10 ppm [27]. The ^1^H NMR spectrum showed peaks in the region of 0.85 to 7.83 ppm (Table 2).

Triterpenes have singlets with chemical shifts of 0.73 to 1.13 ppm, and hydrogens are linked to olefinic carbons (carbons with double bonds) 5.13 and 5.19 [28]. Therefore, the triterpene compounds of the propolis extract, including 24-Norursa-3,12-diene; β-amyrin; Lup-20 (29) -en-3-ol, acetate (3. beta.)- and lanosterol, show these characteristic hydrogen signals. The Lup-20(29) -en-3 beta-ol presents chemical shifts of 16.51 ppm corresponding to carbon 25 (C25); 16.19 ppm (C24); 19.31 ppm (C30); 20.97 ppm (C11); 21.32 ppm (C 2’); 27.46 ppm (C15); 27.09 ppm (C23); 37.11 (C10) [29]. In the expanded spectrum (Supplementary Materials), signals were seen at 16.88 ppm, 19.52 ppm, 21.70 ppm, 21.43 ppm, 27.45 ppm, 27.02 ppm and 37.10 ppm. The compound Lup-20(29) -en-3-ol, acetate (3. beta.), also known as lupeol acetate, presents a pair of singlets with signals of 4.70 and 4.80 ppm for ^1^H–^29^H, which indicates alkene protons in the ^1^H spectrum of the propolis extract at 5.05 and 5.06 ppm [30].

In the carbon NMR spectrum (^13^C NMR), the absence of signals related to functional groups such as ketones and aldehydes that absorb in the range of 192.1 to 203.3 ppm, and carboxylic acid and its derivatives that absorb in the range of 150 to 185, were also observed. The ^13^C NMR spectrum showed signals between 14.40 and 132.08 ppm [27] (Table S1, Figures S2–S5 in Supplementary Materials). The ^1^H NMR and ^13^C NMR spectra confirmed the results obtained in the GC analysis, due to the appearance of signals that correspond to terpenes. The compound 9-octadecenoic acid, methyl ester, (E)- in relation to carbons (CH_2_–CH_2_–CH_2_) shows signals from 29.24 ppm to 32.08 ppm, which is a similar behavior to that which occurs in the compound hexadecanoic acid, methyl ester. Carbon 2 (C-2) and carbon 3 (C-3) show the double bond (C=C) present in the structure of the compound 9-octadecenoic acid, methyl ester, (E)-presents signals at 130 and 129.76 ppm, and in the ^13^C NMR spectrum, signals appeared in these bands (CHEMICAL BOOK).

The compound β-amirin presents a signal with a characteristic displacement of 5.18 ppm (^1^H–^12^H) and 121.70 ppm, which corresponded to 12 C values for the solvent deuterated chloroform (CDCl3) [31]. Therefore, a chemical shift of the respective structure was observed; a carbon is also present in the structure of the compounds lanosterol and 24-Norursa-3,12-diene. Close signals were found in ^1^H NMR and ^13^C NMR. The compound 2-Methyl-5-octyn-4-ol has carbon atoms in its structure with a triple bond (C≡C) that have a chemical shift of 65 to 90 ppm [32]. These triple bonds showed signals with shifts of 72.93 and 73.01 ppm in the ^13^C carbon NMR spectrum. The signal observed at 77.31 ppm is characteristic of a carbon-hydroxyl (C–OH) bond, since this type of carbon can present shifts of 40 to 80 ppm. Carbon 3 (C-3) linked to a hydroxyl group is present in the structures of both triterpene compounds, lanosterol and β-amirin, with a characteristic chemical shift of 78.7 ppm (SpectraBase). Due to the one-dimensional analyses of ^1^H NMR, the ^13^C NMR was carried out with deuterated DMSO solvent, which, as it is a polar aprotic solvent, tends to shift the absorption signals to higher values in relation to the deuterated chloroform solvent (CDCl3).

### 2.3. Physical Appearance of Hydrogels

**Figure 1:**
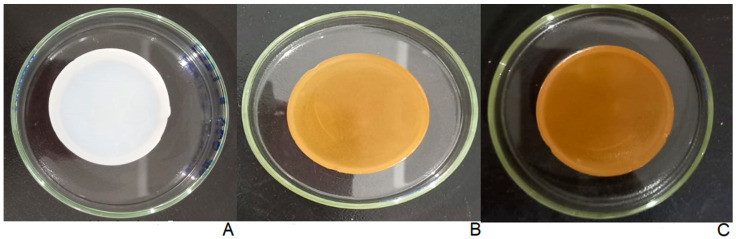
Macroscopic characteristics of hydrogels. (**A**) Blank hydrogel. (**B**) Hydrogel containing 1.0% propolis. (**C**) Hydrogel containing 2.5% propolis.

Hydrogels were examined as to their physical appearance. The hydrogel without extract (blank hydrogel) had a white and translucent color, a typical characteristic of both polyacrylamide and methylcellulose polymers. On the other hand, hydrogels containing 1.0 and 2.5% propolis exhibited a yellowish color, which is characteristic of the propolis extract, with the less concentrated having a lighter color (Figure 1).

### 2.4. Scanning Electron Microscopy (SEM) of Hydrogels

**Figure 2:**
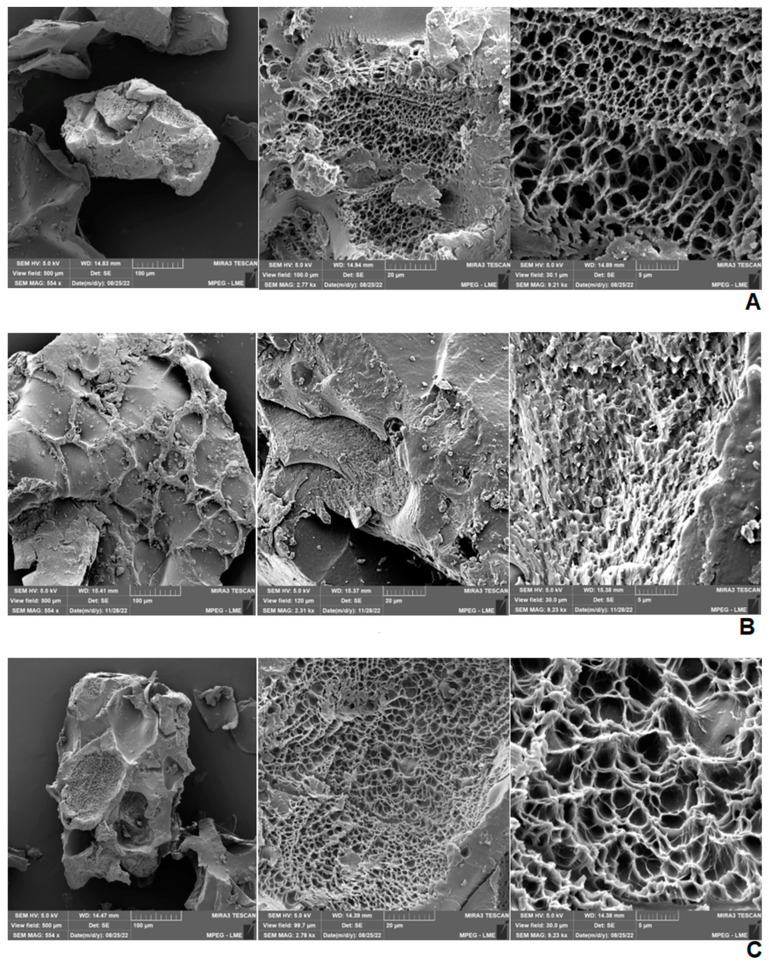
Hydrogel SEM micrographs: (**A**) Blank hydrogel. (**B**) Hydrogel containing 1.0% propolis. (**C**) Hydrogel containing 2.5% propolis. Magnitude: 500×, 2000× and 9000×, respectively.

Hydrogels were examined by Scanning Electron Microscopy (SEM) (Figure 2). The blank hydrogel and the one containing 2.5% propolis showed broad pores, smooth and well-defined walls with variable size, foliaceous structure and presence of the characteristic network of polyacrylamide and methylcellulose hydrogels [33]. It should also be noted that pores were well distributed and interconnected [34] and that the hydrogel containing 2.5% propolis suffered only a small modification in porous structure with a small increase in pore size. On the other hand, in the hydrogel containing 1.0% propolis there was a significant morphological change compared to the plain hydrogel, showing dense and poorly defined pores, almost imperceptible foliaceous distribution, a poorly formed network and layered structure [35]. 

**Figure 3:**
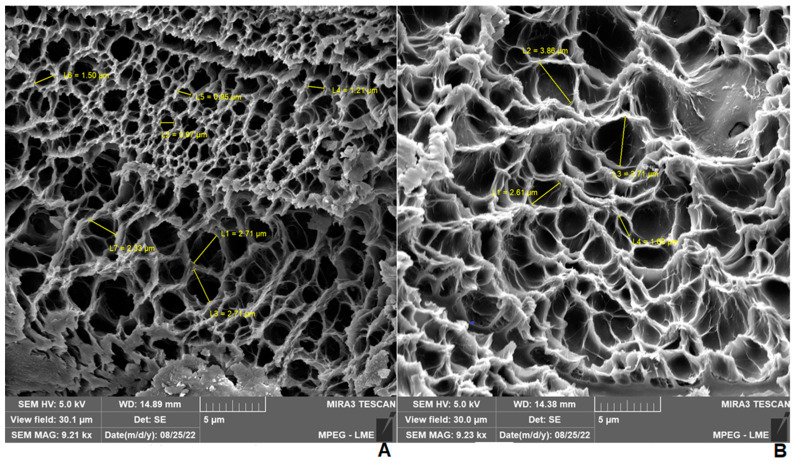
SEM Micrographs of hydrogels at 9000x magnitude with indication of pore size. (**A**) Blank hydrogel. (**B**) Hydrogel containing 2.5% propolis.

The pore size in the blank hydrogel did in fact range from 0.95 to 2.70 µm, while the formulation containing 2.5% extract ranged from 1.68 to 3.86 µm (Figure 3). On the other hand, due to the poor formation of pores in the 1.0% propolis-loaded hydrogel, the size was not able to be estimated.

### 2.5. Swelling Capacity of Hydrogels

The swelling capacity (*Q*) of hydrogels was determined using either phosphate-buffered saline (PBS), a pH 7.4 medium simulating body liquids, or distilled water, for a period of 192 h. 

**Figure 4:**
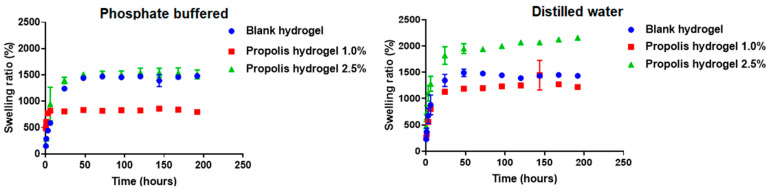
Swelling capacity of blank hydrogel and of hydrogels containing 1.0 and 2.5% propolis in phosphate-buffered saline, pH 7.4, and distilled water.

In PBS, *Q* of all hydrogels was quite high, achieving values as high as 1481, 860 and 1543% for the plain hydrogel and 1.0 and 2.5% for the propolis-containing hydrogels, with equilibrium being reached at 192, 144 and 144 h, respectively (Figure 4). It can be seen that the increase in propolis concentration from 1.0 to 2.5% led to an increase in *Q* of almost 45%, even exceeding the value observed in the blank.

On the other hand, in distilled water, the blank hydrogel swelled up by 1491% and reached equilibrium in 192 h, while hydrogels containing 1.0 and 2.5% propolis swelled up by 1444 and 2092%, reaching equilibrium in 144 and 192 h, respectively (Figure 4). Similar to what happened in PBS, the water absorption capacity of hydrogel containing 2.5% propolis was higher than those of both blank and 1.0% propolis-loaded hydrogels. In summary, the presence of propolis extract increased the swelling capacity of hydrogels.

### 2.6. Mechanical Properties of Hydrogels

**Figure 5:**
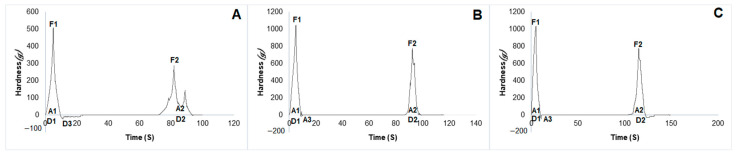
Results of hydrogel Texture Profile Analysis: (**A**) Blank hydrogel. (**B**) Hydrogel containing 1.0% propolis. (**C**) Hydrogel containing 2.5% propolis. F1: strength 1 (1st cycle), hardness. A1: area 1. D1: distance 1. F2: strength 2 (2nd cycle), hardness. A2: area 2. A3: adhesiveness. D2: distance 2. Cohesivity is represented by A1/A2. Elasticity is represented by D2/D1.

The mechanical properties of hydrogels, namely hardness, adhesiveness, stiffness, cohesiveness and elasticity, were evaluated through Texture Profile Analysis (TPA) (Figure 5). Hardness, which was evaluated at the maximum force during compression [36], was significantly higher in the hydrogels containing propolis extract compared to the blank (*p* < 0.01). Adhesiveness, regarded as the work needed to overcome the tensile force between the surface and sample [36], was significantly higher in the hydrogel containing 2.5% propolis, compared to both the blank and the 1.0% propolis hydrogel (*p* < 0.05) (Table 3). This result possibly occurred due to increased internal molecular interactions between extract and polymers. The components of the extract (resins, balsams containing flavonoids, phenolic acids and esters), which have polar characteristics, may have interacted with hydrophilic groups found mainly in methylcellulose and led to stronger interfacial adhesion. In addition, the 1.5% increase in concentration may have further increased these interactions [37]. 

The elasticity property is defined as the ability to stretch the sample and its ability to return to its original size and shape [36] cohesivity is considered to be the work needed to join sample and probe surfaces [36,38] and stiffness is considered to be the ability of a material to resist elastic deformation when subjected to a load or stress [36]. These properties, when evaluated, were significantly higher in the blank hydrogels. The hydrogels containing propolis (*p* < 0.01), however, did not exhibit any statistically significant difference from each other (*p* > 0.01) (Table 3). These results are likely due to the complex structure of the propolis and its high molecular weight. The components of propolis extract, especially those with polar characteristics, may have interacted with polymers and created a crystalline zone, thus reducing hydrogel elasticity, cohesivity and stiffness. Propolis may have also covered the polymeric networks, which may have no longer offered the same resistance to the action of compression force due to lack of contact among them. In addition, the extract may have acted as a dispersed phase, created areas of discontinuity and reduced elasticity and stiffness of the polymeric matrix as well [39]. Another point to consider is the high molecular weight of propolis polyphenols, which, occupying the interchain spaces in the polymeric matrix, may have further reduced these properties [40].

### 2.7. Rheological Behavior of Hydrogels

**Figure 6:**
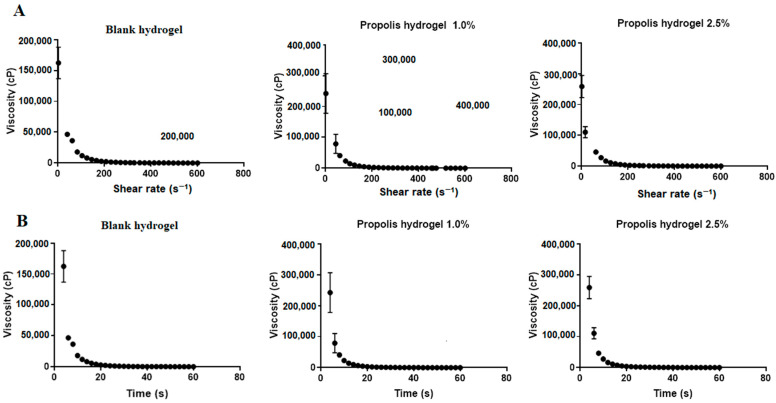
Rheological properties of the blank hydrogel and hydrogels containing 1.0 and 2.5% propolis. (**A**) Viscosity versus shear rate; (**B**) Viscosity versus time.

The rheological properties of hydrogels were evaluated in relation to their tension/deformation and their behavior when exposed to shear stress (*τ*) and shear rate (*γ*). It is known that, as the materials flow, the friction among the molecules present in the material generates a force that gives rise to viscosity and that the greater the friction, the greater the force and the harder it becomes for the material to flow [41]. Therefore, the greater viscosity of hydrogels containing propolis extract reflects greater difficulty in fluidity. Regarding their rheological profile, the hydrogels showed non-Newtonian pseudoplastic behavior, since, as the shear rate increased, viscosity decreased while increasing the interaction among the structural elements in the polymeric matrix. On the other hand, the shear stress increased with the shear rate up to a certain point, beyond which it began to decrease. It was also noted that viscosity decreased over time (Figure 6). 

Table 4 lists the values of viscosity, shear rate and shear stress after 60 s. All formulations showed statistically significantly different viscosity values (*p* < 0.01), but practically coincidental shear rate values (Table 4). It was also observed that the incorporation of the extract into the matrix increased the shear stress, which was much higher (268.36 ± 2.99 Pa) for the hydrogel containing 2.5% propolis compared to both the blank and the 1.0% propolis-containing hydrogels (*p* < 0.01). 

### 2.8. Thermal Behavior of Propolis Extract and Hydrogels by Thermogravimetry (TG/DTG)

Thermogravimetric curves (TG/DTG) of propolis extract, lyophilized and non-lyophilized hydrogels were recorded in order to verify the influence of water on their thermal behavior.

**Figure 7:**
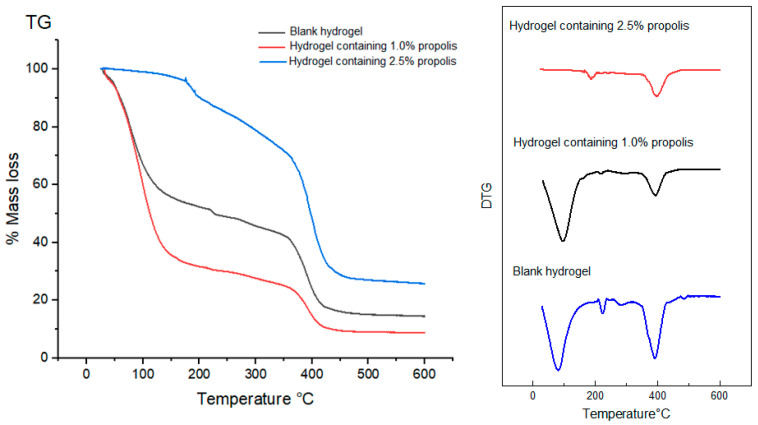
TG/DTG curves of non-lyophilized hydrogels. Conditions: nitrogen atmosphere, flow rate of 50 mL/min, heating rate of 10 °C/min.

The results in Table 5 show that all hydrogels analyzed before freeze-drying showed two peaks of mass loss, the first peak occurring in the range between 40 °C to 170 °C, which would have occurred as a consequence of the evaporation of water contained in the hydrogels, and the second peak within the range of 350 °C to 500 °C, which would be caused by the degradation of the hydrogels’ polymer chains. In particular, in the blank hydrogel the former event occurred in the temperature range 32.02–170.22 °C and the latter in the range 359.57–485.09 °C, with mass losses of 46.55 and 25.95%, respectively (Figure 7. In the 1.0% propolis-containing hydrogel, the two events occurred within the temperature ranges of 41.01–170.92 °C and 365.05–472.71 °C, with mass losses of 62.42 and 14.46% (Figure 7), while in the 2.5% propolis-containing one they occurred in the ranges 44.22–173.00 °C and 195.27–570.50 °C, with mass losses of 4.02 and 64.81%, respectively (Figure 7).

**Figure 8:**
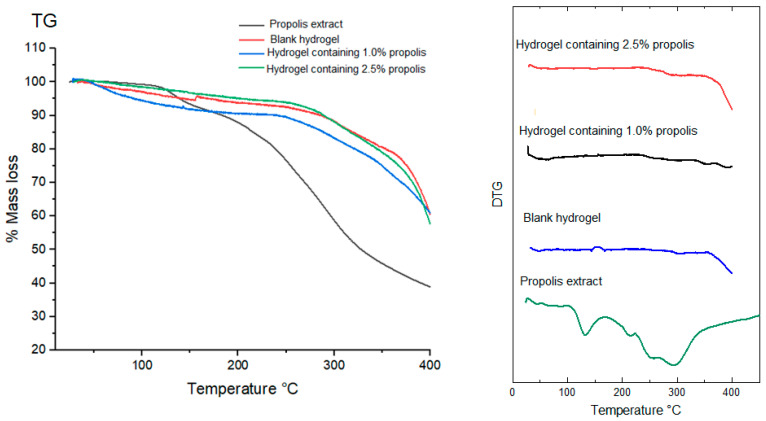
TG/DTG curves of lyophilized hydrogels and propolis extract. Conditions: nitrogen atmosphere, flow rate of 50 mL/min, heating rate of 10 °C/min.

While two mass loss events were observed in non-lyophilized hydrogels, the lyophilized hydrogels showed only one mass loss event, within the range of 280 °C to 400 °C, with no events occurring before 200 °C. Specifically, the lyophilized blank hydrogel showed a mass loss event (Figure 8) at a range of 379.68–391.45 °C, with loss of 17.65%. The hydrogels containing 1.0 (Figure 8) and 2.5% propolis (Figure 8) showed mass losses within the temperature ranges 352.56 °C–392.12 °C and 287.10–393.11 °C, with 25.37 and 27.06% losses, respectively (Figure 8). This difference in the thermal profile between the hydrogels before and after freeze-drying is explained by the water content present in those not freeze-dried. In the composition of hydrogels there is 90% water; this water is used in the formation of the hydrogel during polymerization, and during the storage of the hydrogel this water is lost to the environment. What free water remains evaporates when the non-lyophilized hydrogel sample is subjected to TG analysis. Finally, thermogravimetry is very sensitive to water content; this was evidenced in our results, in which samples with water in their composition had completely different thermograms than those of the dried samples. Finally, in the TG/DTG curves of propolis extract, two mass loss events are observed. The first event occurred in the range of 124.57 °C–146.59 °C, the second mass loss event occurred between 268.52 °C–341.47 °C (Figure 8).

In all hydrogels, either lyophilized or not, the degradation event occurred between 300 and 400 °C (Table 5).

### 2.9. Thermal Behavior of Propolis Extract and Hydrogels by Differential Scanning Calorimetry (DSC)

Differential Scanning Calorimetry (DSC) analyses were also performed on propolis extract, lyophilized and non-lyophilized hydrogels in order to better elucidate their thermal behavior. 

**Figure 9:**
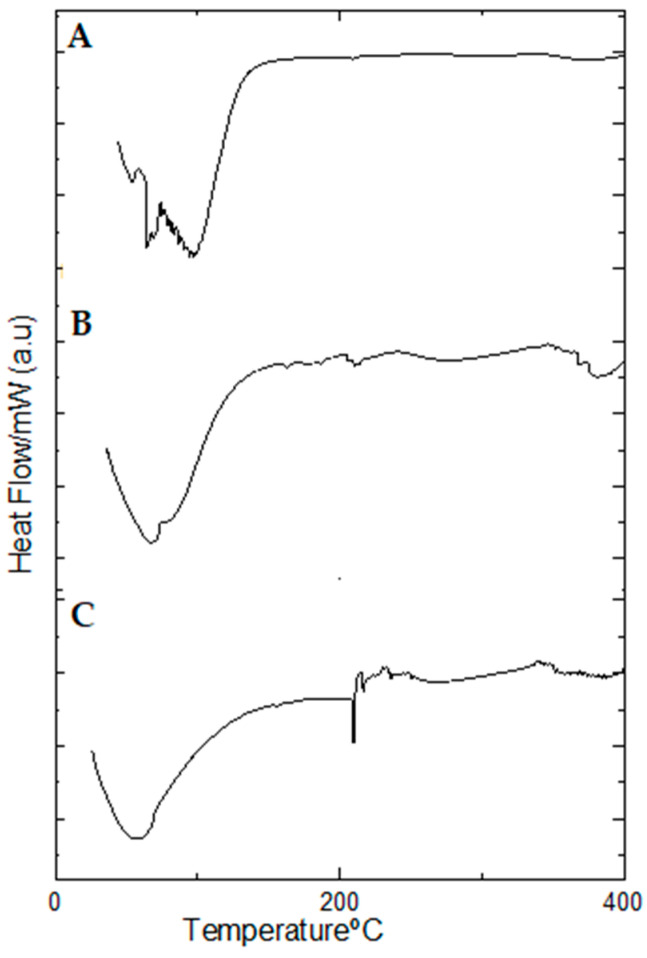
DSC curves of non-lyophilized hydrogels: (**A**) Hydrogel containing 2.5% propolis. (**B**) Hydrogel containing 1.0% propolis. (**C**) Blank hydrogel. Conditions: nitrogen atmosphere, flow rate of 50 mL/min, heating rate of 10 °C/min.

In the DSC curves of the non-lyophilized hydrogels (Figure 9), there only appears an endothermic event related to water evaporation, which occurred within the temperature ranges between 38.12–107.85 °C, 43.14–108.16 °C and 48.77–121.02 °C for the blank hydrogel and 1.0 and 2.5% propolis hydrogels (Figure 9A–C), respectively.

**Figure 10:**
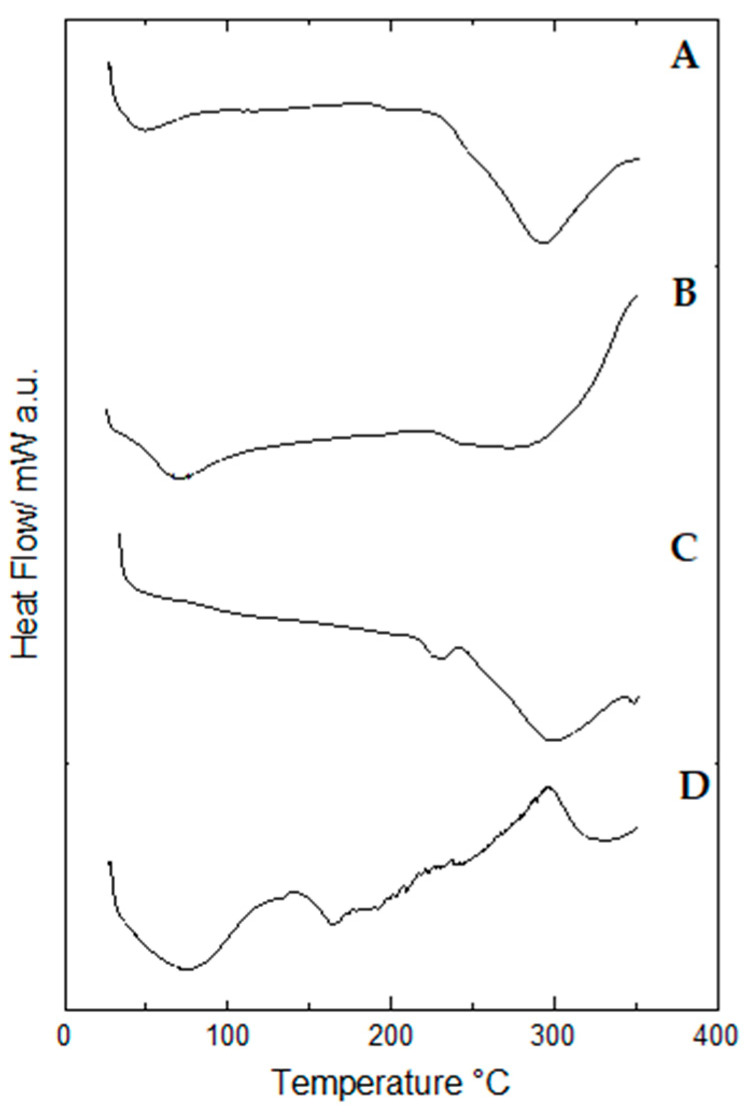
DSC curves of lyophilized hydrogels: (**A**) Hydrogel containing 2.5% propolis. (**B**) Hydrogel containing 1.0% propolis. (**C**) Blank hydrogel. (**D**) Propolis extract. Conditions: nitrogen atmosphere, flow rate of 50 mL/min, heating rate of 10 °C/min.

However, in the DSC curves of propolis extract (Figure 10) three endothermic events occurred. The first event occurred in the range between 78.80–156.79 °C, with a peak temperature at 126.94 °C, with the second event, with a peak temperature at 195.94 °C, occurring in the range between 194.71–207.45 °C, while the third event occurred in the range of 205.86–217.84 °C with a peak temperature at 215.53 °C.

Lyophilized hydrogels showed only one endothermic event for the blank (at 247.40–339.00 °C) (Figure 10B) and two endothermic events for the propolis hydrogels 1% (at 48.45–95.56 °C and 244.62–330.55 °C) (Figure 10C) and 2.5% (at 32.13–73.66 °C and 236.70–33.16 °C) (Figure 10D).

Table 6 lists the values of peak temperature and enthalpy variation (Δ*H*) of the heat exchange events which occurred in both lyophilized and non-lyophilized hydrogels.

### 2.10. Fourier-Transform Infrared (FTIR) Spectroscopy Profiles of Propolis Extract and Hydrogels

**Figure 11:**
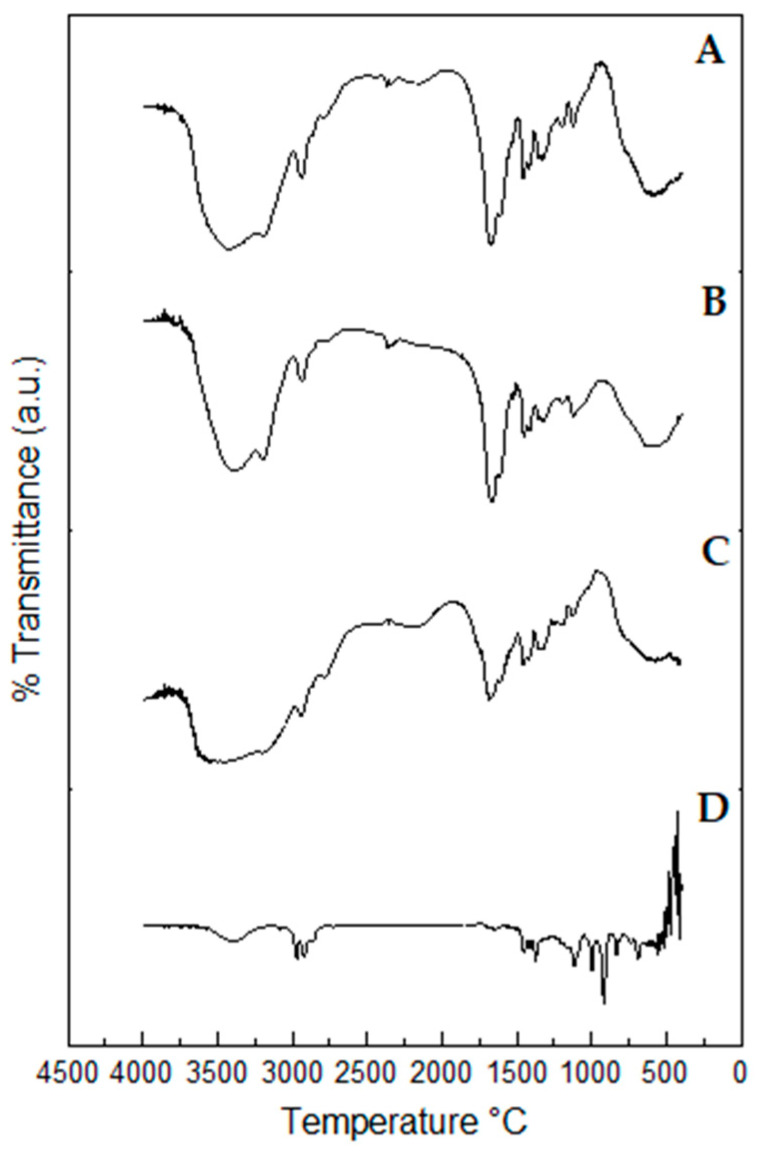
FTIR spectra of lyophilized hydrogels: (**A**) Hydrogel containing 2.5% propolis. (**B**) Hydrogel containing 1.0% propolis. (**C**) Blank hydrogel. (**D**) Propolis extract. The analyses were performed in the wavenumber range of 4000 to 400 cm^−^^1^, with 32 scans and resolution of 4 cm^−^^1^.

Fourier-Transform Infrared (FTIR) spectra provide evidence of the presence of functional groups in the structure of chemical compounds [42], which can be used to characterize pharmaceutical inputs and extracts in formulation and pre-formulation studies [42]. Fourier-Transform Infrared (FTIR) spectroscopy profiles of the propolis extract and hydrogels are shown in Figure 11.

The spectra of hydrogels containing 1.0 and 2.5% propolis showed a band related to axial deformation vibration of N –H bond in amines (at 3431 and 3423 cm^−^^1^, respectively), a band related to symmetrical axial deformation vibration of N –H bond in primary amide (at 3198 and 3175 cm^−^^1^, respectively), a band referring to the axial deformation vibration of C –H bond in methyl groups (at 2926 and 2950 cm^−^^1^, respectively), a peak related to axial deformation of C=O bond in amides (both at 1670 cm^−^^1^), an absorption band related to angular deformation of C –H bond in cycloalkanes (both at 1460 cm^−^^1^), a band referring to angular deformation vibration of O –H in primary alcohols (at 1305 and 1329 cm^−^^1^, respectively), and a peak corresponding to axial deformation vibration of C –O bond in alcohols (at 1119 and 1111 cm^−^^1^, respectively) (Figure 11B,C) [43].

The blank spectrum showed a band at 3493 cm^−^^1^ related to axial deformation vibration of N –H bond in amines, an absorption peak at 2950 cm^−^^1^ referring to axial deformation vibration of C –H bond in methyl groups, a peak at 1670 cm^−^^1^ related to axial deformation of C=O bond in amides, an absorption band at 1460 cm^−^^1^ related to angular deformation of C –H bond in cycloalkanes, an absorption peak at 1336 cm^−^^1^ referring to angular deformation vibration of O –H bond in primary alcohols and an absorption peak at 1134 cm^−^^1^ corresponding to axial deformation vibration of C –O bond in alcohols (Figure 11C) [43].

One can observe in the extract spectrum a band at 3398 cm^−^^1^, related to axial deformation vibration of O –H in alcohol; a band at 3085 cm^−^^1^, showing to axial deformation of C –H in alkenes; a band at 2968 cm^−^^1^, referring to axial deformation of C –H in alcohol; a band at 2924 cm^−^^1^, showing to symmetric axial deformation of C –H in methylene groups; a band at 2855 cm^−^^1,^ showing the axial vibration deformation of C –H in methylene groups; a 1644 cm^−^^1^ band, referring to axial deformation vibration of C=C in alkenes; a 1450 cm^−^^1^ band, related to asymmetric angular deformation of C –H in methyl groups; a band at 1375 cm^−^^1^, related to symmetric angular deformation of C –H bonds in methyl group; a 1112 cm^−^^1^ band, related to axial deformation vibration of C –O in alcohol; a band at 995 cm^−^^1^, which refers to the out-of-plane angular deformation of C-H in alkenes; a 918 cm^−^^1^ band, showing the angular deformation of C –H in isopropyl groups; the band at 835 cm^−^^1^, which shows the out-of-plane angular deformation vibration of C –H in polynuclear aromatic hydrocarbons, and the band at 688 cm^−^^1^ relating to the out-of-plane symmetric angular deformation in amide N –H groups (Figure 11D) [42].

A great similarity was noticed between the FTIR spectra of the blank hydrogel and the hydrogels loaded with propolis extract. Nonetheless, the incorporation of 1.0 and 2.5% propolis in the hydrogels led to an increased intensity of peaks at 1662 –1670 cm^−^^1^ and 1453 –1460 cm^−^^1^, which can be ascribed to vibration of C=C bonds in aromatic rings possibly resulting from functional groups of secondary metabolites contained in the extract. On the other hand, all blank hydrogel bands were preserved in the spectra of propolis-loaded hydrogels, which demonstrates not only the extract incorporation but also the hydrogel’s compatibility with the extract [44]. The spectra obtained in both hydrogels correspond to bands related to both polyacrylamide and methylcellulose, as well as bands found in the propolis chemical structure [45]. 

### 2.11. Total Polyphenol and Flavonoid Contents of Propolis Extract and Hydrogels

Hydrogels are known to be excellent systems for the release of both lipophilic and hydrophilic drugs or active substances, which makes them the target of several studies [46]. Extracts from natural products can be incorporated into the matrix of hydrogels to release their active compounds, including phenolics [47]. In this study, phenolic compounds were extracted from the hydrogel matrix and determined spectrophotometrically in the propolis extract and hydrogels. As expected, the hydrogel incorporating the extract at the higher concentration had higher contents of both total polyphenols and flavonoids (*p* < 0.01) (Table 7). 

### 2.12. Antioxidant Activity of Propolis Extract and Hydrogels

**Figure 12:**
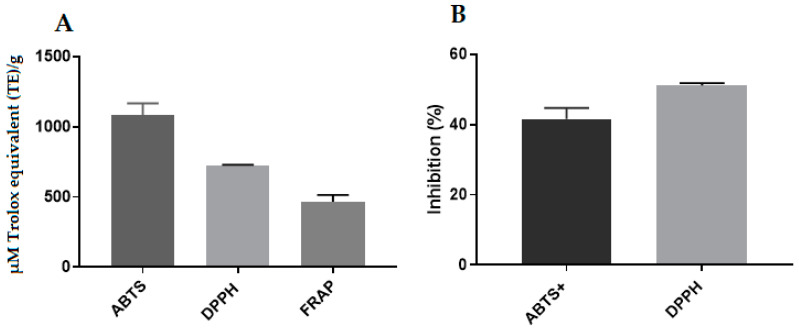
Values of the antioxidant activity of propolis extract, by the ABTS, DPPH and FRAP assays. (**A**) Expressed in µM Trolox equivalent (TE)/g. (**B**) Percentage of inhibition.

**Figure 13:**
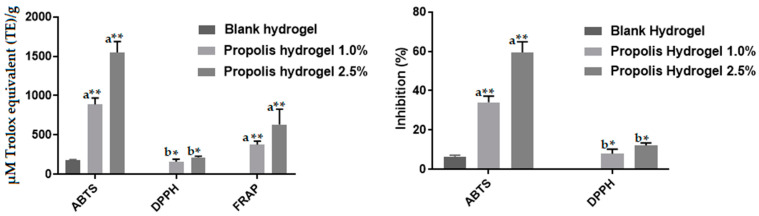
Values of the antioxidant activity of blank and propolis-containing hydrogels determined by the ABTS, DPPH and FRAP assays. Different letters in the same column indicate significant difference. ^a^** (*p* < 0.01) and ^b^* (*p* < 0.05).

The antioxidant activity of the propolis extract and hydrogels was determined by the DPPH, ABTS and FRAP assays. In particular, the values determined by the DPPH and ABTS assays were expressed in µM Trolox equivalent (TE)/g and percentage of inhibition, while those determined by the FRAP assay only in µM Trolox. Although propolis extract (Figure 12) and both hydrogels containing propolis (Figure 13) had good antioxidant activity as determined by the three methods, the more concentrated one showed significantly higher values (*p* < 0.05 by the DPPH and *p* < 0.01 by the ABTS and FRAP assays) due to higher concentration of antioxidant substances [48].

The blank hydrogel showed antioxidant activity only by the ABTS assay (Figure 13); however, its value was much lower than those of hydrogels containing the extract because it contained only the polymers and reagents used to form its network.

### 2.13. Biocompatibility of Propolis Extract and Hydrogels 

**Figure 14:**
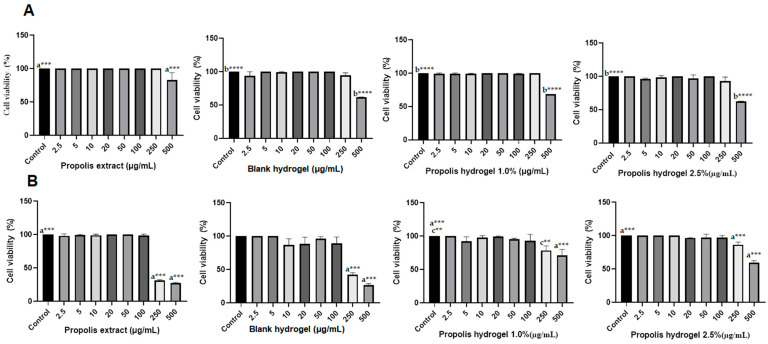
Viability of (**A**) Vero fibroblast-like kidney cells and (**B**) macrophages (J774) after 24 h of treatment with different extract and hydrogel concentrations (2.5, 5, 10, 20, 50, 100, 250 and 500 μg/mL). Control: Without treatment. The results were analyzed by the analysis of variance (ANOVA) followed by the Tukey test, ^b^**** *p* < 0.0001, ^a^*** *p* < 0.001, ^c^** *p* < 0.01.

Biocompatibility of the extract and propolis-loaded hydrogels was evaluated in terms of viability of fibroblast cells (Vero) (Figure 14A) and macrophages (Figure 14B) at different concentrations (2.5–500 µg/mL) using the MTT assay. 

In the extract, only the concentration of 500 µg/mL showed a significant reduction when compared to the control, but did not reduce cell viability up to 50% in cells of the fibroblast lineage (CC50 > 500 µg/mL) (Figure 14A). However, in relation to macrophage cells, there was a significant reduction in cell viability, starting at a concentration of 250 µg/mL (Figure 14B). Only the 500 µg/mL concentration in both hydrogels showed significant reduction in Vero cells viability compared to the control, but without reaching the value of 50%. In particular, the hydrogels containing 1.0 and 2.5% propolis and the blank hydrogel led to 31, 38 and 40% viability reductions, respectively (Figure 14A). Although none of the hydrogels showed cytotoxicity in the concentration range 2.5–100 µg/mL against macrophage cells, at concentrations of 250 and 500 µg/mL the blank led to 50% and <50% cell viability reductions, the hydrogel containing 1.0% propolis to 22 and 29% reductions, and that containing 2.5% propolis to 14 and 41% reductions, respectively (Figure 14B).

## 3. Discussion

Wound treatment is still a challenge faced by health systems worldwide. Despite the development of numerous biomaterials used as dressings, there are several factors that can make the healing process difficult such as diabetes and wound contamination by microbial agents [49]. Wounds that do not heal or are slow to heal are a serious problem, and for this reason there is an urgent need for innovative therapies that address this issue [20]. Conventional dressings become critical in healing chronic wounds resulting from slow-healing tissue lesions [50]. The use of natural extracts in new dressings can facilitate the healing process due to their biological properties, such as antibacterial, anti-inflammatory, antifungal and antioxidant activities [51]. 

Propolis is a natural product with potential for use in active dressings due to its proven therapeutic properties [26]. Modern wound dressings obtained from biomaterials are biocompatible, biodegradable and non-toxic [52]. With this in mind, hydrogels are an excellent alternative bandage material, due to the fact that their porous interconnected matrix mimics the physicochemical properties of the tissue environment and can absorb biological fluids [34]. 

It is therefore necessary to perform quality control of antioxidant biomaterials developed with the aim of employing them as dressings for wound healing. As a result, polyacrylamide/methylcellulose hydrogels incorporating propolis extract in their matrix were prepared in this work and tested for their main quality, safety and effectiveness parameters as well as antioxidant potential, as a potential application as a dressing in the treatment of wounds.

To this end, Scanning Electron Microscopy (SEM) was used as a tool to examine the microstructural characteristics (morphology) of polymeric hydrogels [53]. Micrographs showed porous structures connected with each other either in the blank hydrogel or the hydrogels containing propolis extract in two different concentrations, consistently with the recognized capacity of porous materials to form gels in contact with biological fluids [52]. It was also observed that the incorporation of the extract led to pore filling. Very porous dressings such as the hydrogels prepared in this study are known to facilitate cell growth and assist in tissue regeneration. SEM evidenced well-defined network and three-dimensional structure of the blank hydrogels and 2.5% propolis-loaded hydrogels. Reticulation is of great importance in obtaining polymeric networks that determine the internal structure of hydrogels and improve mechanical properties, molecule transport and swelling behavior [34]. 

The ability to swell is another fundamental property of hydrogels, especially those used as dressings for wounds, which must have the ability to absorb excess exudate and keep the site of the wound humid and hydrated, in addition to allowing proper permeability of oxygen [54]. Hydrogels showed high ability to swell in both distilled water and phosphate-buffered saline (PBS), probably due to the presence of hydrophilic groups of methylcellulose as well as hydroxyl groups of phenolic compounds present in the extract [55]. The high swelling capacity in both water and PBS of the blank and 2.5% propolis-containing hydrogels, i.e., those that showed a well-defined network in SEM micrographs, suggests that hydrogel swelling was favored by reticulation. On the other hand, the hydrogel containing 1.0% propolis showed a poorly defined network and lower swelling capacity, probably due to a space reduction among polymeric chains resulting from increased reticulation density [56]. The high swelling capacity of propolis-loaded hydrogels reveals how beneficial they could be to the wound bed, as it suggests their potential to absorb the exudate [57].

Linked to this context, mechanical properties are of great importance in hydrogels, especially when used as coverage in the treatment of wounds, because good mechanical resistance, flexibility and elasticity contribute to maintaining the integrity of skin tissue when damaged by external forces, thus becoming a protection for the wound [58]. The hydrogels prepared in this study proved to be adhesive, a feature that could be explored to fix the dressing in the appropriate place over the period required to treat the wound [36]. It is also expected that, thanks to their flexibility, they would adapt well to the injury surface and allow smoother contact, adaptation to skin irregularities and adjust to different areas, thus ensuring more efficient and uniform coverage [59]. Proper hardness of hydrogels offers the advantage of maintaining their structure, increasing stability, and less susceptibility to deformations when applied to the skin, as well as ensuring prolonged maintenance of a moist and favorable wound bed, which is paramount in the healing process [60]. 

The poor cohesivity of hydrogels suggests slight resistance to breakage or disintegration, which, on the contrary, would be necessary to preserve structure and provide efficient and continuous coverage necessary for healing [61]. However, their good elasticity suggests that, when subjected to certain external forces, they would return to their original structure, a characteristic capable of ensuring protection to the wound bed [62]. Although the blank hydrogel had greater elasticity than the others, all hydrogels showed viscoelastic behavior, which could ensure some wound protection in case of external shocks.

Regarding rheological properties, which influence important parameters such as spreadability, syringeability and release of drug or other substances [63], hydrogels had non-Newtonian behavior, i.e., their viscosity was dependent on shear rate [36]. As for flow, they exhibited pseudoplastic behavior, i.e., direct proportionality between shear rate and shear stress, although not continuously, as well as decreased viscosity that allows the material fluidity when subjected to friction [64]. Pseudoplastic behavior is considered a desirable property in products intended for topical use, as the formulation should spread properly during application, flow easily after applying tension, form a uniform film on the skin [65] and remain in place without flowing, once applied [66]. It can be the result of the well-organized network structure formed by hydrogen bonds and polymer entanglement, which ensures high viscosity at low shear rates and prevents dispersed phase wetting [64]. Finally, the rheological behavior of hydrogels allows to better understand the physicochemical phenomena related to spreadability and grip on the skin [64,66], and plays an essential role in their adhesive properties and can influence skin regeneration [67]. 

Thermogravimetry (TG/DTG) and Differential Scanning Calorimetry (DSC) were used to evaluate the thermal behavior of hydrogels. Thermal analyses are a set of techniques extensively used in the development of drugs, which provide information on the thermal stability, crystallinity and melting point of materials [68]. Specifically, thermal stability of hydrogels is required to face their sterilization by moist or dry heat before application to the wound bed. All TG/DTG curves of non-lyophilized hydrogels showed two main mass loss events, the former due to evaporation of water present in the formulations and the latter to thermal degradation of components at temperatures above 200 °C (Table 3). Instead, lyophilized hydrogels exhibited only one mass loss event and similar TG/DTG curves. This result may have been due to the poor sensitivity of thermogravimetry to minor modifications in sample composition like those induced by propolis incorporation in a much smaller proportion than that of hydrogel-forming polymers. This also suggests that propolis did not change the physicochemical characteristics of hydrogels in which it was incorporated, as expected for any drug delivery system [42]. Similar to the TG/DTG curves, the DSC curves were affected by the amount of water contained in the samples. Non-lyophilized hydrogels showed a main initial event, with wide and strong peak resulting from water evaporation, while the lyophilized ones showed a well-defined endothermic event above 200 °C probably due to degradation of hydrogel polymeric chains [14]. 

Propolis is a complex mixture containing a variety of secondary metabolites [69], such as phenolic compounds that contain hydroxyl groups and aromatic rings in simple or polymeric form [70]. Phenolic compounds, especially flavonoids, have adequate structure for radical scavenging, which makes them more effective antioxidants than vitamins C and E [71]. The strong antioxidant activity of plant extracts and apiculture products has been associated with their high contents especially of flavonoids [72]. Most flavonoids have biological activities, such as the antioxidant, anti-inflammatory and antibacterial ones, which are of great importance to the food, cosmetic and pharmaceutical sectors [73] and have aroused interest in the discovery of new natural compounds capable of minimizing oxidative damage to cells [48]. The contents of total polyphenols and total flavonoids found in the hydrogels prepared in this study prove their importance as possible dressings.

In this context, active dressings should exert antioxidant properties, avoid excessive oxidation, regulate inflammation and assist in repairing wounds [74]. Excess production of free radicals in response to tissue damage complicates the healing process, since free radicals are capable of destroying proteins, lipids and extracellular matrix [46]. Active hydrogels have the ability to scavenge reactive oxygen species (ROS) without causing harmful effects [75], restrain cell damage caused by oxidative stress and free radicals to the wound site, and therefore promote healing. In addition, due to their gelling properties, they are suitable materials to cover the irregular area of the wound [75]. Antioxidant hydrogels are divided into three subgroups: (a) hydrogels whose matrix has antioxidant properties, (b) hydrogels loaded with antioxidant agents, and (c) hydrogels containing multifunctional antioxidants [76]. Hydrogels used in the present study can fit both categories of hydrogels with antioxidant properties, as their base (blank hydrogel) displayed antioxidant activity by the ABTS assay, and hydrogels loaded with antioxidant agents, since the two hydrogels containing propolis showed high antioxidant activity by the DPPH, FRAP and ABTS assays. It is also noteworthy that extract incorporation led to an increase in the ABTS antioxidant activity of hydrogels. Moreover, their high antioxidant activity assessed by all methods confirms their possible use as an alternative dressing for wound treatment, given the importance of antioxidant activity in the healing process.

The in vitro cytotoxicity test is the first test to evaluate the biocompatibility of any material for use in biomedical devices [77], and the good biocompatibility of hydrogel dressings is paramount in for use in clinical applications [78]. Cytocompatibility is a preliminary analysis necessary to validate the application potential of hydrogels in the biomedical area as dressing for wounds and skin repair materials, as they prepare the site for cell growth [79]. In this study, cell viability was evaluated in fibroblast and macrophage cells, which are both involved in the healing stage. In particular, the main function of fibroblasts is to maintain tissue integrity by the synthesis of extracellular matrix components [80]. The test of Vero fibroblast cells treated with the hydrogels for 24 h showed that there was no change in cell viability in the concentration range of 2.5–250 µg/mL compared to the control group without treatment, which indicates that the tested substance had no cytotoxic effect under these conditions. Only the concentration of 500 µg/mL showed moderate cytotoxicity. Macrophages are inflammatory cells recruited to the wound site that persist at all phases of the repair response [81]. They are very important cells for the healing process because they participate in phagocytosis, bacterial cell death and cell debris removal, in addition to being the main sources of cytokines and growth factors that stimulate fibroblast proliferation [82]. Cell viability of macrophage cells after 24 h of exposure was reduced only at concentrations of 250 µg/mL or higher. In general, hydrogels containing the propolis extract can be considered safe until concentrations of 250 µg/mL, and they constitute a valid alternative for use as a dressing in wound healing.

## 4. Materials and Methods

### 4.1. Chemicals and Reagents

Acrylamide ≥ 99% (AAm), sodium persulfate ≥ 98% (SP), methylcellulose with 15 cP viscosity (MC), N,N,N,N-tetramethylethylenediamine 99% (TEMED), N,N′-methylenebisacrylamide 99% (MBAAm), gallic acid, quercetin, 2,2-diphenyl-1-picrylhydrazyl (DPPH), 2,2-azinobis (3-ethylbenzothiazoline-6) sulfonic acid (ABTS), 6-hydroxy-2,5,7,8-tetramethylchroman-2-carboxylic acid (Trolox) and 3-(4,5-dimethylthiazol-2-yl)-2,5-diphenyltetrazolium bromide (MTT) were purchased from Sigma-Aldrich (St. Louis, USA); trihydrate sodium acetate, hexahydrate ferric chloride, heptahydrate ferrous sulfate, 2,4,6-tris(2-pyridyl)-s-triazine (TPTZ), sodium carbonate, aluminum chloride and potassium bromide were purchased from LabSynth (São Paulo, Brazil).

### 4.2. Propolis Samples

Propolis samples were provided by Embrapa Amazônia Oriental (Belém, Brazil). Samples of the bee species, *Apis mellifera* L., were collected at Sitio Melmel, located in the municipality of Capitão Poço, state of Pará, northern region of Brazil (geographic coordinates: latitude of 43°40′0″ and longitude of 47°40′1″) on 20 January 2022, in two different types of hives of the same product, in vegetation with different plant species. At the time of collection, the day was cloudy and humid, over a 12 h period. The samples were divided into small portions, packed into plastic bags and kept under refrigeration. The coarse propolis was shredded in a crusher (Scientific equipment S.A., São Paulo, Brazil) to reduce particle size.

### 4.3. Preparation of the Propolis Extract

The crushed propolis (200 g) was subjected to maceration in 2 L of ethanol/water solution (70:30, *v*/*v*) in an amber flask, protected from light, at room temperature for 7 days. The resulting tincture was filtered and evaporated using a rotavap (Buchi R-210, Geneva, Switzerland) at 40 °C up to full evaporation of the solvent. The concentrated propolis extract was then packed in amber bottles and kept under refrigeration. Part of the extract was subjected to drying in a freeze-drier (Liotop L101, Liobras, São Carlos, Brazil) for subsequent analyses [22].

### 4.4. Gas Chromatography-Mass Spectrometry Assay (GC-MS) of Propolis Extract

Chemical characterization was carried out on a gas chromatograph with a QP 2010 Plus mass selective detector (Shimadzu^®^, Kyoto, Japan), in electron impact ionization mode (70 eV), using a splitless injector. Before injection into the chromatograph, the sample was prepared using a mixture of 250 mg of the extract added to 5 mL of methanolic NaOH solution. Fifteen mL of the esterification reagent, containing 2 g of ammonium chloride, 60 mL of methanol and 3 mL of sulfuric acid heated for 15 min, was added. After this period, it was heated again at reflux for another 3 min and transferred to a separation funnel with 25 mL of hexane and 50 mL of ultrapure water. After separation of the phases, the aqueous phase was discarded and the organic phase was collected for injection into the gas chromatograph. The operating conditions were as follows: capillary column: RTX5MS 30 m, 0.25 mm × 0.25 μm thick, program temperature: 100 to 300 °C at a rate of 3 °C/min; division rate: 1/80; T execution: 66 min; carrier gas: helium (1 mL/min); injector temperature: 250 °C; interface temperature: 260 °C; scan mode: 40–800 (Daltons) and injection volume: 1 μL [83].

### 4.5. Nuclear Magnetic Resonance Spectrometry (NMR) of Propolis Extract

The propolis extract at a concentration of 20 mg was solubilized in 600 µL of deuterated dimethyl sulfoxide (DMSO-D6). The **^1^**H and **^13^**C spectra were obtained on a Bruker spectrometer (model Ascend™ 400MHz, Boston, MA, USA). The TopSpin 3.6.2 software was used for control and data processing, with manual calibration by suppressing the residual H_2_O signal, and adjusting the **^1^**H and **^13^**C spectra by the residual solvent signal, DMSO-H6, respectively, at 2.49 ppm and 39.5 ppm [84].

### 4.6. Preparation of Propolis-Containing Hydrogels

Hydrogels were prepared by chemical polymerization, via free radical, using 7.2% (*w*/*v*) AAm, 0.5% (*w*/*v*) MC and 8.55 µmol/mL MBAAm as a reticulation agent. Then, the propolis extract was added to the reaction medium at a concentration of 1.0 or 2.5%, together with 3.38 µmol/mL SP as initiator of the polymerization reaction and 3.21 µmol/mL TEMED as a catalyst. The hydrogel without the extract obtained under the same conditions was used as a blank. Next, nitrogen was bubbled in the solution for 20 min with the purpose of removing oxygen until completing polymerization [15]. Finally, hydrogels were washed in distilled water for 3 days with water change every 24 h and then lyophilized (Liotop L101, Liobras, São Carlos, Brazil).

### 4.7. Scanning Electron Microscopy (SEM) of Hydrogels

The morphological examination of hydrogels was performed using a scanning electronic microscope (Mira 3, Tescan, Brno-Kohoutovice, Czech Republic). Dry powdered hydrogels were deposited on stubs containing carbon tape. The surface was metallized with a thin layer of gold/palladium to allow the electrical conductivity necessary for the formation of images, which were obtained with magnitude of 500×, 2000× and 9000× [6].

### 4.8. Swelling Capacity of Hydrogels

Swelling capacity of hydrogels was determined either in distilled water or in phosphate-buffered saline (PBS), pH 7.4. Dry hydrogels were weighed and immersed in 100 mL of the selected medium at 37 °C. Samples were collected at fixed time intervals (0.5, 1, 3, 6, 24, 48, 72, 96, 120, 144, 168 and 192 h) and, after removing excess fluid absorbed on their surface with filter paper, they were weighed on an analytical balance. The swelling capacity (*Q*) was calculated by Equation (1) [6]:

(1) $$Q=\frac{W_{s}-W_{d}}{W_{d}}\times 100$$

where *W*_d_ is the mass of swollen hydrogel and *W*_s_ that of dry hydrogel.

### 4.9. Mechanical Properties of Hydrogels

Texture Profile Analysis (TPA) of the formulations was performed on a texture analyzer (TA-XTplus, Stable Micro Systems, Godalming, UK). For this purpose, the equipment was placed in compression mode, and samples were subjected to double compression by a 10 mm diameter probe, at a fixed rate of 2 mm/s and a depth of 10 mm, with a delay period of 15 s between the first compression and the beginning of the second compression. Hardness, adhesiveness, stiffness, elasticity and cohesiveness were the parameters evaluated. The analyses were performed in triplicate [36].

### 4.10. Rheological Properties of Hydrogels

The rheological properties of hydrogels were determined using an RST rheometer (Brookfield, Middleboro, MA, USA) with an RCT-50-1 spindle, turning at 100 rpm at fixed temperature (37 °C) for 60 s. The analyses were performed in triplicate [36]. 

### 4.11. Thermal Behavior by Thermogravimetry (TG/DTG) of Propolis Extract and Hydrogels

The TG/DTG curves of both lyophilized and non-lyophilized hydrogels and extract were obtained in a TGA-50 thermal analyzer (Shimadzu, Kyoto, Japan), using an aluminum crucible containing 3.0 mg of sample, under nitrogen (N_2_) atmosphere and 50 mL/min flow rate. The analyses were conducted within the temperature range of 25 to 600 °C for non-lyophilized hydrogels and 25 to 400 °C for lyophilized hydrogels, using a heating rate of 10 °C/min. Data were analyzed with the TA-50W software, version 2.21 (Shimadzu, Kyoto, Japan) [14]. 

### 4.12. Thermal Behavior by Differential Scanning Calorimetry (DSC) of Propolis Extract and Hydrogels

The DSC curves of both lyophilized and non-lyophilized hydrogels and extract were obtained in a DSC-60 Plus equipment (Shimadzu, Kyoto, Japan). Samples (2.0 mg) were placed in an aluminum crucible, and the analyses were performed in N_2_ atmosphere at a flow rate of 50 mL/min, a heating rate of 10 °C/min and a temperature of 550 °C [14]**.**

### 4.13. Fourier-Transform Infrared (FTIR) Spectroscopy Profile of Propolis Extract and Hydrogels

The lyophilized hydrogels and extract (1 mg) were mixed with 0.99 mg of potassium bromide under high pressure to form pads. The absorption spectra were obtained in a spectrometer (IR Prestige-21, Shimadzu) in the wavenumber range from 4000 to 400 cm^−1^, with 32 scans and resolution of 4 cm^−1^ [14]. The spectroscopic profile was obtained using the Origin Pro, version 9.0 2019 software (OriginLab).

### 4.14. Extraction of Phenolic Compounds from the Hydrogel Matrix

The phenolic compounds were extracted from the hydrogel matrix by adding 2 mL of ethanol solution to 0.1 g of powdered hydrogel. The solution was homogenized in vortex for 1 min, placed in an ultrasound bath (CD 4820, Cleaner Kondentech, São Paulo, Brazil) for 20 min and centrifuged (Eppendorf 5804 R, Hamburg, Germany) for 15 min at 7500 rpm. Finally, the supernatant was filtered in membranes with 0.45 µm pore diameter (Millipore, Bedford, MA, USA) [85].

#### 4.14.1. Determination of Total Polyphenol Content of Propolis Extract and Hydrogels

Total polyphenol content was determined in a UV 1800 spectrophotometer (Shimadzu, Kyoto, Japan) at 760 nm wavelength using a standard gallic acid curve at concentrations in the range 5–75 mg/mL. After mixing 100 μL of the hydrogel solution and extract, 500 μL of the Folin–Ciocalteu reagent and 6 mL of distilled water in a 10 mL volumetric flask, the resulting mixture was left to rest for 2 min. Then, 2 mL of 20% (*w*/*v*) sodium carbonate solution were added, the resulting mixture was mixed for 30 s and the volume was completed with distilled water. After resting for 2 h, readings were performed at 760 nm. Results were expressed in milligrams of gallic acid equivalent per gram of extract (mg GAE/g) [86].

#### 4.14.2. Determination of Total Flavonoid Content of Propolis Extract and Hydrogels

Total flavonoid content was determined in the same UV 1800 spectrophotometer (Shimadzu, Kyoto, Japan) at 425 nm wavelength using a standard quercetin curve at concentrations in the range 5–30 mg/mL. After mixing 800 μL of the hydrogel suspension and extract with 1 mL of 2.5% (*w*/*v*) aluminum chloride solution in a 10 mL volumetric flask, the volume was completed with ethanol. The mixtures remained at rest for 30 min, and then readings were performed at 425 nm. Results were expressed in milligrams of quercetin equivalent per gram of extract (mg QE/g) [86].

### 4.15. Antioxidant Activity Determination of Propolis Extract and Hydrogels

#### 4.15.1. ABTS Assay

Antioxidant activity by capturing the ABTS^.+^ radical cation was determined using the same UV 1800 spectrophotometer (Shimadzu, Kyoto, Japan) mentioned above at 734 nm wavelength. The ABTS^.+^ solution was prepared by reaction of 5 mL of 7 mM ABTS solution with 88 µL of 140 mM potassium persulfate solution and kept at room temperature in the dark for 16 h. In a dark environment, a 30 µL aliquot of hydrogel suspension and extract was transferred to test tubes and mixed with 3.0 mL of ABTS^.+^ solution. Then the mixture was homogenized in vortex for 6 min, and readings were performed at 734 nm. Ethanol was used as a blank to calibrate the spectrophotometer. The antioxidant activity was calculated based on a standard Trolox curve at concentration in the range 100–2000 µM and expressed in µM Trolox equivalent (TE)/g. The inhibition percentage was calculated according to Equation (2) [87]:

(2) $$\%\;inhibition=\frac{\left(Abs\;control-Abs\;sample\right)}{Abs\;control}\times 100$$

where Abs,c is control absorbance and Abs,s is sample absorbance.

#### 4.15.2. DPPH Assay

Antioxidant activity by capturing the DPPH^.^ free radical was determined in the same spectrophotometer mentioned above at 515 nm wavelength. Then, a 150 µL aliquot of hydrogel suspension and extract was transferred to test tubes containing 5 mL of DPPH^.^ work solution, and the resulting mixture was homogenized in vortex. After 30 min, readings were performed at 515 nm. The antioxidant activity was calculated based on a standard Trolox curve at concentration in the range 50–2000 µM and expressed in µM Trolox equivalent (TE)/g. The inhibition percentage was calculated according to Equation (2) [88]. 

#### 4.15.3. FRAP Assay

Antioxidant activity by the Ferric Reducing Antioxidant Power (FRAP) assay was determined in the same spectrophotometer mentioned above at 595 nm wavelength. In a dark environment, a 90 μL aliquot of hydrogel suspension and extract was transferred to a test tube, and 270 μL of distilled water and 2.7 mL of the reaction solution were added. After homogenization in vortex and heating in a water bath at 37 °C for 30 min, readings were performed at 595 nm. The reaction solution was used as a blank to calibrate the spectrophotometer. The antioxidant activity was calculated based on a standard Trolox curve at concentrations in the range 160–1600 µM and expressed in µM TE/g [89]. 

### 4.16. In Vitro Hydrogel and Extract Biocompatibility

#### 4.16.1. Cultivation of Fibroblast and Macrophage Cells

Vero fibroblast-like kidney cells and macrophage cells (J774), which were used to evaluate the cytotoxicity of hydrogels [90], were purchased from the Rio de Janeiro Cell Bank and kept at 37 °C in a humidified atmosphere with 5% CO_2_. The culture medium was the Dulbecco’s Modified Eagle Medium (DMEM) complemented with 10% fetal bovine serum. Culture media were renewed until the formation of a cell monolayer. Cells were washed with PBS, trypsinized, centrifuged for 10 min at 1200 rpm and then counted in a Neubauer chamber. Cells at the concentration of 1 × 10^4^ cells/mL of DMEM were distributed in 96-well plates and incubated for 24 h at 37 °C in 5% CO_2_/95% air for stabilization [91]. 

#### 4.16.2. Cell Viability Analysis 

For the analysis of cell viability by the MTT assay, 1 × 10^4^ Vero and macrophage cells per mL were cultivated in 96-well plates and subjected to treatment with hydrogels and extract at concentrations of 2.5, 5, 10, 20, 50, 100, 250 and 500 μg/mL at 37 °C for 24 h in a greenhouse containing 5% CO_2_. The supernatant was then removed, the wells were washed with PBS, and MTT (0.5 mg/mL) diluted in PBS was added. After incubation at 37 °C in an incubator containing 5% CO_2_ for 3 h, the supernatant was removed, the wells were washed with PBS, and 200 μL of DMSO were added to each well to solubilize the formazan crystals under agitating incubation for 10 min. Subsequently, the final solution was transferred to 96-well plates and read in a spectrophotometer (BIO-RAD Model 450 Microplate Reader, São Paulo, Brazil) at 570 nm wavelength. Cells killed with 15% formaldehyde were used as control [91].

### 4.17. Statistical Analysis

Cell viability and swelling capacity were analyzed using GraphPad 5.0. For statistical evaluation, the analysis of variance (ANOVA) and the Tukey test were used, considering statistically significant variations corresponding to *p* < 0.05 or *p* < 0.01, depending on the analyzed parameter. All analyses were performed in triplicate.

## 5. Conclusions

In this study, hydrogels containing propolis extract, in two different concentrations, were prepared and tested for the purpose of using them as a dressing for wound healing. The chemical characterization of the extract showed the presence of phenolic substances, including flavonoids, using the UV-visible technique, and terpenes, identified by GC-MS and ratified by NMR, in addition to showing good thermal stability using thermal analysis techniques. The FTIR spectra showed an aggregate of bands belonging to important functional groups such as alcohol, methyl and methylene and aromatic groups. The hydrogels (both the blank and those containing concentrations of propolis extract) were obtained and showed a characteristic appearance of the polymers and propolis extract; a three-dimensional network and a well-defined porous structure were also observed. The hydrogels showed a high capacity to swell both in water and in a medium that simulates biological fluids (PBS), an important characteristic for biomaterials to be used as dressings. As for mechanical properties, they proved to be adhesive, cohesive and elastic. Its rheological properties were compatible with biomaterials exhibiting non-Newtonian pseudoplastic fluid behavior. The extract and hydrogels (both the blank and those containing concentrations of propolis extract) did not show a cytotoxic effect up to a concentration of 250 µg/mL on fibroblast cells. However, in macrophage lines, there was a reduction in cell viability from a concentration of 250 µg/mL. The extract and hydrogels containing propolis extract showed good antioxidant activity by the three methods analyzed. Therefore, hydrogels containing propolis extract, due to their antioxidant properties, low cost and easy availability, from a technological point of view, can constitute a promising alternative as a covering for the treatment of skin lesions.

## Figures and Tables

**Table 1:** Compounds and terpenes identified in propolis extract by Gas Chromatography (GC-MS).

Identified Compounds	Retention Time	Area %
Pentan-2-ol, 4-allyloxy-2-methyl-	2.070	4.77
Hexane, 1-(3-butenyloxy)	2.170	2.49
2-Pentene, 4,4-dimethyl-, (Z)-	2.284	2.71
Cyclopropane, 1,1,2,3-tetrameth	2.480	0.94
2-Cyclopenten-1-one, 3-methyl-	2.535	1.99
2-Methyl-5-octyn-4-ol	2.538	0.72
2-Hexanone, 6-hydroxy-	2.640	0.82
1,4-Hexadiene, 4-methyl-	2.962	1.32
Hexadecanoic acid, methyl ester	29.305	11.25
9,12-Octadecadienoic acid (Z,Z)	34.683	1.48
9-Octadecenoic acid, methyl ester	34.879	32.20
Methyl stearate	35.696	1.92
Docosanoic acid, methyl ester	47.031	1.98
Tetracosanoic acid, methyl ester	52.084	1.79
Terpenes		
Lanosterol	48.073	6.00
β-Amyrin	49.608	9.83
24-Norursa-3,12-diene	50.459	17.13
Lup-20(29)-en-3-ol, acetate(3. beta.)-	52.400	7.33
Thunbergol	66.174	2.29

**Table 2:** Chemical shifts and multiplicity observed in the ^1^H NMR spectrum of propolis extract.

RMN ^1^H			
Chemical Shift δ (ppm)	Multiplicity	Type of Proton	Class
0.85	Singlet	CH_3_	Methyl
1.23	Singlet	CH_3_	Methyl
1.55	Singlet	CH_2_	Methylene
1.63	Singlet	CH_2_	Methylene
1.99	Singlet	(C=C–C–H)	Methylene
2.17	Singlet	(C=C–C–H)	Methylene
2.50	Singlet	(C=C–H) sp^2^	DMSO
5.05	Duplet	(C=C–H) sp^2^	Alkenes
5.32	Singlet	(C=C–H) sp^2^	Alkenes
6.58	Singlet	(C=C–H) sp^2^	Alkenes
6.82	Duplet	(C=C–H) sp^2^	Alkenes/aromatics
7.83	Duplet	(C=C–H) sp^2^	Alkenes/aromatics

**Table 3:** Mechanical properties of blank hydrogel and of hydrogels containing 1.0 and 2.5% propolis.

	Cycle 1			Cycle 2	
Formulation	Hardness (g)	Adhesiveness (mJ)	Stiffness (mm)	Cohesiveness (Dimensionless)	Elasticity (mm)
Blank hydrogel	415.57 ± 131.79 ^a^**	0.52 ± 0.37 ^b^*	2.03 ± 0.37 ^a^**	0.98 ± 0.01 ^a^**	31.55 ± 18.88 ^a^**
Hydrogel containing 1.0% propolis	1430.70 ± 185.83 ^a^**	0.83 ± 0.12 ^b^*	0.62 ± 0.14 ^a^**	0.82 ± 0.15 ^a^**	9.96 ± 0.13 ^a^**
Hydrogel containing 2.5% propolis	1337.13 ± 377.13 ^a^**	1.44 ± 0.51 ^b^*	0.80 ± 0.17 ^a^**	0.95 ± 0.24 ^a^**	10.41 ± 0.60 ^a^**

Different letters in the same column indicate significant difference. ^a^** difference of hydrogel containing propolis extract compared to the blank (*p* < 0.01). ^b^* difference of hydrogel containing 2.5% propolis compared to both the blank and the 1.0% propolis-containing hydrogel (*p* < 0.05).

**Table 4:** Values of the rheological properties of the blank hydrogel and the hydrogels containing 1.0 and 2.5% propolis after 60 s.

Formulation	Viscosity (cP)	Shear Rate (s^−1^)	Shear Stress (Pa)
Blank hydrogel	108.44 ± 10.15 ^a^**	601.84 ± 0.031	63.10 ± 6.97 ^b^**
Hydrogel containing 1.0% propolis	149.50 ± 21.92 ^a^**	601.92 ± 0.012	90.01 ± 13.19 ^b^**
Hydrogel containing 2.5% propolis	445.67 ± 5.18 ^a^**	601.68 ± 0.106	268.36 ± 2.99 ^b^**

Different letters in the same column indicate significant difference. ^a^** difference of hydrogel containing propolis extract compared to the blank (*p* < 0.01). ^b^** difference of hydrogel containing 2.5% propolis compared to both the blank and the 1.0% propolis-containing hydrogel (*p* < 0.01).

**Table 5:** Mass losses detected in propolis extract and hydrogels by thermogravimetric analysis and related temperature ranges.

	Sample	1st Stage		2nd Stage	
		Temperature Range (°C)	Mass Loss (%)	Temperature Range (°C)	Mass Loss (%)
	Propolis extract	124.57–146.59	7.35	268.52–341.47	44.92
Non-lyophilized samples	Blank hydrogel	32.02–170.22	46.55	359.57–485.09	25.95
	Hydrogel containing 1.0% propolis	41.01–170.92	62.42	365.05–472.71	14.46
	Hydrogel containing 2.5% propolis	44.22–173.00	4.02	195.27–570.81	38.37
Lyophilized samples	Blank hydrogel	379.68–391.45	17.65	–	–
	Hydrogel containing 1.0% propolis	352.56–392.12	25.37	–	–
	Hydrogel containing 2.5% propolis	287.10–393.11	27.06	–	–

**Table 6:** Peak temperatures and enthalpy variations of heat exchange events which occurred in propolis extract, blank hydrogel and hydrogels containing 1.0 and 2.5% propolis.

		1st Event		2nd Event		3rd Event	
	Sample	Peak Temperature (°C)	Enthalpy Variation (J/g)	Peak Temperature (°C)	Enthalpy Variation (J/g)	Peak Temperature (°C)	Enthalpy Variation (J/g)
	Propolis extract	74.8	−43.76	327.4	−1.02	215.53	−2.48
Non-lyophilized samples	Blank hydrogel	54.71	−222.66	-	-		
	Hydrogel containing 1.0% propolis	67.92	−502.27	-	-		
	Hydrogel containing 2.5% propolis	97.13	−1.52	-	-		
Lyophilized samples	Blank hydrogel	299.66	−152.45				
	Hydrogel containing 1.0% propolis	70.71	−119.52	273.37	−397.26		
	Hydrogel containing 2.5% propolis	43.31	−33.24	293.28	−311.88		

**Table 7:** Contents of phenolic compounds determined by UV-Vis spectrophotometry in propolis extract and 1.0 and 2.5% propolis-containing hydrogels.

Sample	Total Polyphenols (mg GAE/g)	Total Flavonoids (mg QE/g)
Propolis extract	72.80 ± 1.20	35.19 ± 0.24
Blank hydrogel	-	-
Hydrogel containing 1.0% propolis	24.74 ± 1.71 ^a^**	8.01 ± 0.99 ^a^**
Hydrogel containing 2.5% propolis	32.10 ± 1.01 ^a^**	13.81 ± 0.71 ^a^**

Experiments were performed in triplicate, and data are presented as mean values and standard deviation. Results were analyzed using ANOVA and Tukey test, ^a^** *p* < 0.01.

## Data Availability

All the data are presented in this work.

## Supplementary Materials

The following supporting information can be downloaded at: https://www.mdpi.com/article/10.3390/ph17050575/s1, Figure S1: **^1^**H NMR spectrum of propolis extract from the bee *Apis mellifera* L.; Figure S2: **^13^**C NMR spectrum of propolis extract from the bee *Apis mellifera* L.; Figure S3: Expanded **^13^**C NMR spectrum of *Apis mellifera* bee propolis extract between the regions of 40 to 14 ppm; Figure S4: Expanded **^13^**C NMR spectrum of propolis extract between the regions of 77 to 63 ppm; Figure S5: Expanded **^13^**C NMR spectrum of propolis extract between the regions of 132 to 115 ppm.
