# Supplementary material for: Characterization, Biocompatibility and Antioxidant Activity of Hydrogels Containing Propolis Extract as an Alternative Treatment in Wound Healing
Source: Pharmaceuticals (Basel). 2024 Apr 30;17(5):575. doi: 10.3390/ph17050575 (PMC11123975; doi:10.3390/ph17050575)
Supplement: Supplementary file 1 [file pharmaceuticals-17-00575-s001.zip › pharmaceuticals-2946730-supplementary.pdf]

Supplementary material

Table S1: Chemical shifts and multiplicities observed in the ^{13}C NMR spectrum of propolis extract.

RMN ^{13}C			
Chemical shift δ (ppm)	Multiplicity	Type of carbon	Class
14.40	Singlet	$R{-}CH_3$, $R_2{-}CH_2$ and $R_3{-}CH$	Hydrocarbon
16.56	Singlet	$R{-}CH_3$, $R_2{-}CH_2$ and $R_3{-}CH$	Hydrocarbon
17.97	Singlet	$R{-}CH_3$, $R_2{-}CH_2$ and $R_3{-}CH$	Hydrocarbon
18.20	Singlet	$R{-}CH_3$, $R_2{-}CH_2$ and $R_3{-}CH$	Hydrocarbon
19.52	Singlet	$R{-}CH_3$, $R_2{-}CH_2$ and $R_3{-}CH$	Hydrocarbon
21.42	Singlet	$R{-}CH_3$, $R_2{-}CH_2$ and $R_3{-}CH$	Hydrocarbon
22.55	Singlet	$R{-}CH_3$, $R_2{-}CH_2$ and $R_3{-}CH$	Hydrocarbon
23.83	Singlet	$R{-}CH_3$, $R_2{-}CH_2$ and $R_3{-}CH$	Hydrocarbon
24.95	Singlet	$R{-}CH_3$, $R_2{-}CH_2$ and $R_3{-}CH$	Hydrocarbon
25.92	Singlet	$R{-}CH_3$, $R_2{-}CH_2$ and $R_3{-}CH$	Hydrocarbon
27.23	Singlet	$R{-}CH_3$, $R_2{-}CH_2$ and $R_3{-}CH$	Hydrocarbon
28.35	Singlet	$R{-}CH_3$, $R_2{-}CH_2$ and $R_3{-}CH$	Hydrocarbon
29.48	Singlet	$R{-}CH_3$, $R_2{-}CH_2$ and $R_3{-}CH$	Hydrocarbon
31.76	Singlet	$R{-}CH_3$, $R_2{-}CH_2$ and $R_3{-}CH$	Hydrocarbon
33.82	Singlet	$R{-}CH_3$, $R_2{-}CH_2$ and $R_3{-}CH$	Hydrocarbon
34.12	Singlet	$R{-}CH_3$, $R_2{-}CH_2$ and $R_3{-}CH$	Hydrocarbon
37.10	Singlet	$R{-}CH_3$, $R_2{-}CH_2$ and $R_3{-}CH$	Hydrocarbon
39.29-40.54	Septet	DMSO	
63.65	Singlet	C≡C	Alkyne
65.94	Singlet	C≡C	Alkyne
67.33	Singlet	C≡C	Alkyne
70.50	Singlet	C≡C	Alkyne
71.02	Singlet	C≡C	Alkyne
72,.3	Singlet	C≡C	Alkyne
73.01	Singlet	C≡C	Alkyne
77.31	Singlet	C−OH	Alkyne
115.43	Singlet	C=C	Alkyne
124.57	Singlet	C=C	Alkyne
130.11	Singlet	C=C	Alkyne
132.08	Singlet	C=C	Alkyne

Figure S1: ^{1}H NMR spectrum of propolis extract from the bee *Apis mellifera* L.

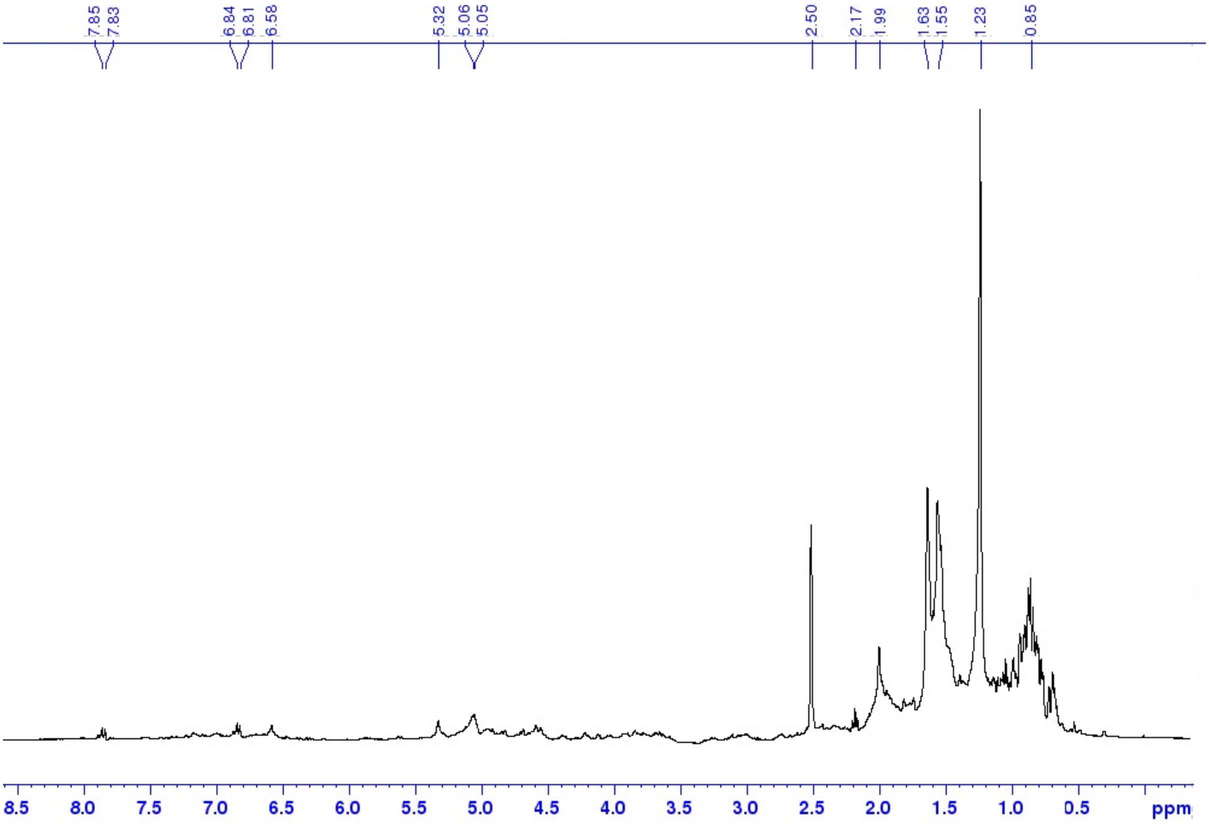

Figure S2: ^{13}C NMR spectrum of propolis extract expanded in the regions between 132.08 and 14.40 ppm.

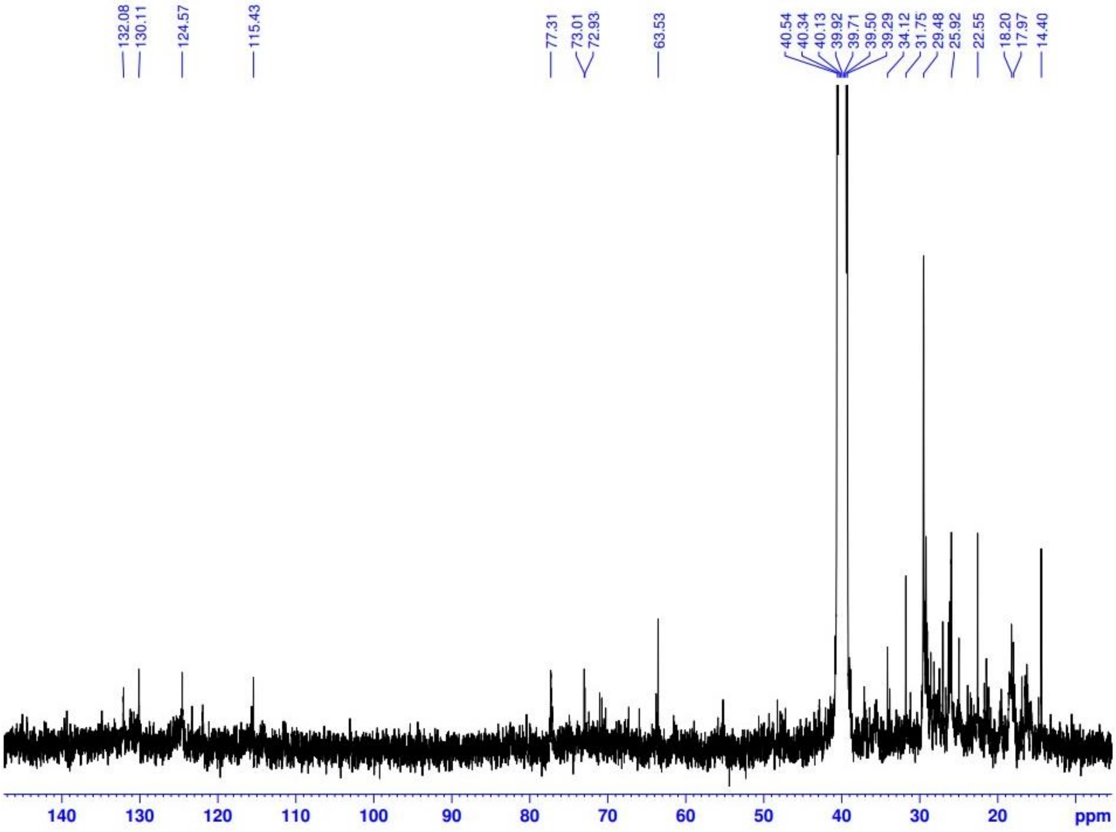

Figure S3: Expanded ^{13}C NMR spectrum of *Apis mellifera* bee propolis extract between the regions of 40 to 14 ppm.

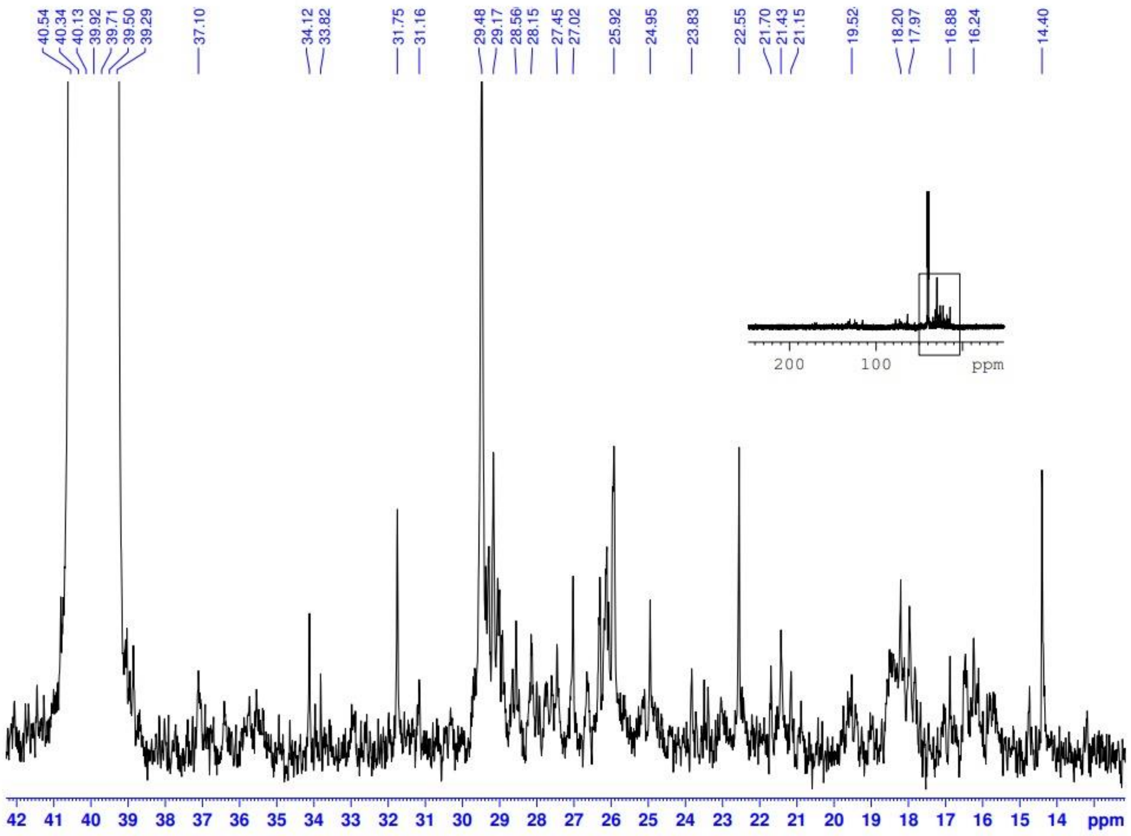

Figure S4: Expanded ^{13}C NMR spectrum of *Apis mellifera* bee propolis extract between the regions of 77 to 63 ppm.

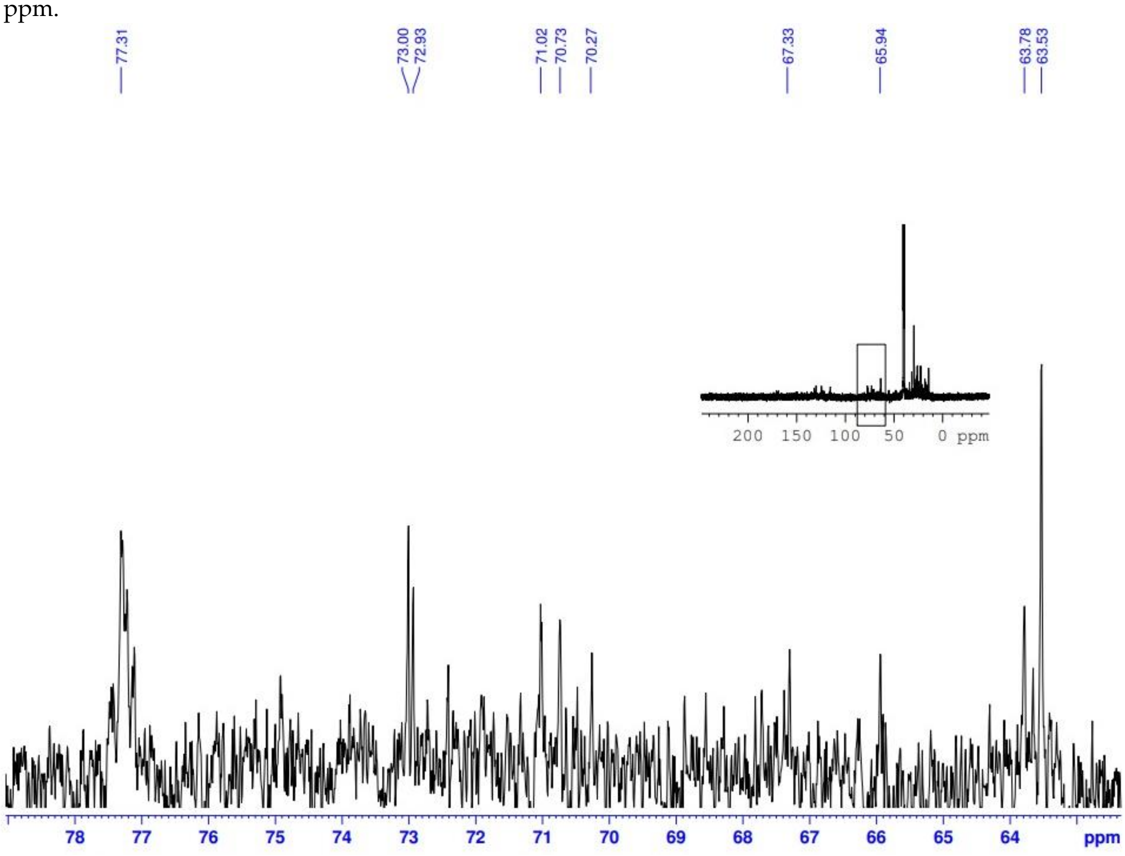

Figure S5: Expanded ^{13}C NMR spectrum of propolis extract from the bee *Apis mellifera* between the regions of 132 to 115 ppm

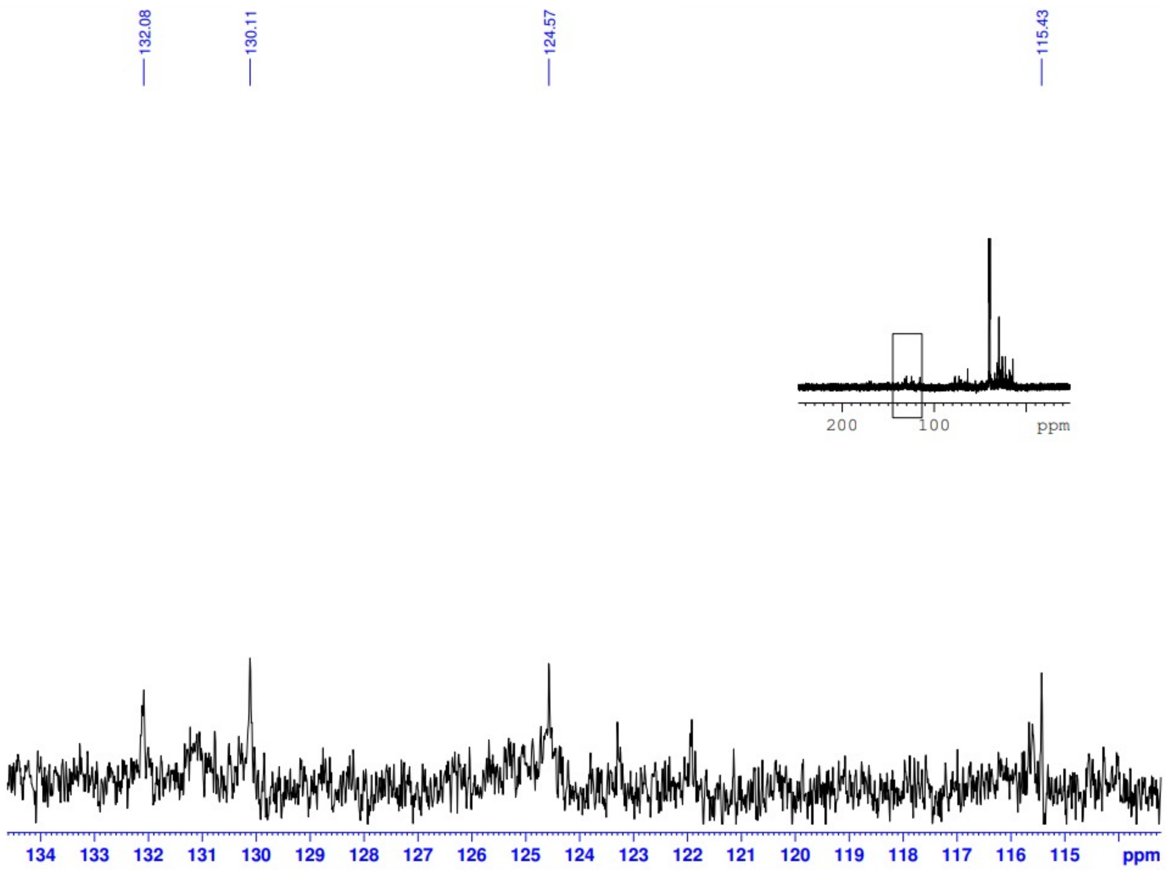
132.08
130.11
124.57
115.43
200
100
ppm
134 133 132 131 130 129 128 127 126 125 124 123 122 121 120 119 118 117 116 115
ppm
